# A Survey of Deep Learning-Based Image Restoration Methods for Enhancing Situational Awareness at Disaster Sites: The Cases of Rain, Snow and Haze

**DOI:** 10.3390/s22134707

**Published:** 2022-06-22

**Authors:** Sotiris Karavarsamis, Ioanna Gkika, Vasileios Gkitsas, Konstantinos Konstantoudakis, Dimitrios Zarpalas

**Affiliations:** Visual Computing Lab, Information Technologies Institute, Centre for Research and Technology Hellas (CERTH), 57001 Thessaloniki, Greece; skaravarsamis@iti.gr (S.K.); ioanna.gkika@iti.gr (I.G.); gkitsasv@iti.gr (V.G.); k.konstantoudakis@iti.gr (K.K.)

**Keywords:** deraining, dehazing, desnowing, deep learning, deep neural networks

## Abstract

This survey article is concerned with the emergence of vision augmentation AI tools for enhancing the situational awareness of first responders (FRs) in rescue operations. More specifically, the article surveys three families of image restoration methods serving the purpose of vision augmentation under adverse weather conditions. These image restoration methods are: (a) deraining; (b) desnowing; (c) dehazing ones. The contribution of this article is a survey of the recent literature on these three problem families, focusing on the utilization of deep learning (DL) models and meeting the requirements of their application in rescue operations. A faceted taxonomy is introduced in past and recent literature including various DL architectures, loss functions and datasets. Although there are multiple surveys on recovering images degraded by natural phenomena, the literature lacks a comprehensive survey focused explicitly on assisting FRs. This paper aims to fill this gap by presenting existing methods in the literature, assessing their suitability for FR applications, and providing insights for future research directions.

## 1. Introduction

First responders (FRs) are trained professionals that provide aid in case of emergency and include medics, firefighters, law enforcement and civil protection officials. During operations, FRs often come across many stressful situations, experience extreme safety risks and may meet an additional inevitable obstacle—adverse weather conditions. Weather conditions that reduce visibility, such as rain, snow or haze, prevent FRs from exhibiting expected performance under normal conditions while they simultaneously increase the risk of injury. Moreover, adverse weather conditions may become an obstacle on the performance of any computer vision (CV) tool employed by FRs during operations. Typically, one would observe a degraded performance in these tools that handle raw visual data that is itself degraded by artifacts caused by the utilization of the tools under adverse weather conditions.

In recent years, thanks to deep learning (DL), CV algorithms have been proposed for solving various vision tasks which, in the real world, are either difficult, time consuming or impossible for a human to carry out. In such tasks, DL-based CV algorithms have shown impressive results. Due to their great performance, recent works have concentrated on developing them further and making them more accurate and possibly faster. To make a DL model more accurate, many researchers have focused on designing sophisticated architectures. This led to the requirement of training large models that can achieve performance close to that of humans, but with such a computational complexity that would be impossible to be implemented in devices with constrained resources or in applications in which CV algorithms need to perform in real time. This challenge led many scientists to research ways to achieve outstanding performance with models that contain a considerably low number of parameters. At the same time, evolution in hardware has given us the ability to use numerous sensors, processors and energy sources in small and lightweight gadgets. As a consequence, portable devices have paved the way for extending CV applications in numerous domains that require the existence of light equipment and quick response from the model. These devices can serve the enhancement of the FRs’ vision.

On the other hand, the development and deployment of CV systems for facilitating the FRs’ vision under adverse weather conditions (herein, we focus on rain, snow and haze) pose several challenges that need to be addressed. First, there is a lack of assortment in the imagery provided by datasets which are focused on a specific task. Although there are several tasks directly related to the enhancement of visual recognition under adverse conditions (such as deraining, desnowing and dehazing), virtually none of them contains scenes representative of the FRs’ working environment. Additionally, even in the design of the existing datasets, it is impossible for the designer to simultaneously capture the same image with and without the visual artifacts caused by the adverse weather conditions. However, being able to do so is a requirement in standard supervised end-to-end approaches that receive pairs of clear and noisy images. In such models, the noisy image is fed at one end of the model and the clean image is output at the other end. Thus the models proposed in the literature are either trained in an unsupervised manner having only the noisy images, or they use pairs of synthetic noisy images with their ground truth (clear) images. A direct drawback when training models on synthetic data, however, is that there is a domain gap among the real and the synthetic datasets. That is, in such a case, it may be difficult for image denoising models to generalize well on real-world data.

Another important issue in the application of CV systems for the facilitation of the FRs’ vision, is the required processing time. When employing a DL model to enhance the situational awareness of FRs, the model should process visual data in near real-time and often in infrastructure-less environments (e.g., environments with an absence of network connectivity and with power constraints). In the related literature, only a small number of research works propose deep neural network models that are lightweight architectures able to operate well in power-constrained infrastructures. Under these conditions, there is a requirement for the DL-based augmented vision tools to simultaneously be lightweight and adequately accurate.

Owing to the constraint on real-time processing and the requirement for lightweight models, there is an urgent need for solutions that can handle more than one task simultaneously. These solutions are often called unified solutions, and we will be using this term in our article. So far, there has been a great amount of research works on image restoration techniques focusing on only one specific adverse weather condition. In the real world, however, during a rescue operation (e.g., one taking more than one day to complete) the FRs could meet any weather condition. This means that the CV tool that could help the FRs’ vision should be able to adapt to the visibility changes of the scene, or it should be able to switch between modes of operation specific to one particular weather condition. Importantly, the unification of such models has already been addressed in the literature (at least regarding the three problems that this article is concerned with). Hence, to the best of our knowledge, there are a few models that suggest unified solutions.

In ideal weather conditions, CV tools can offer the means for FRs to keep themselves safe and at the same time locate and save victims robustly and reliably. However, rain, snow and haze can degrade the performance of such CV systems. Consequently, these systems do not provide optimal assistance to FRs. In this survey article, we explore the use of DL-based image denoising methods tailored towards the removal of atmospheric artifacts in captured images. These DL models could be applied for the augmentation of the FRs’ vision. We specifically require these inferences to be better possible under rain, snow and haze conditions.

Clear vision can improve disaster response in multiple ways, including damage detection [1], traffic management [2,3], UAV-driven reconnaissance [4,5] and flood detection [6]. Object detection is a computer vision task which is usually impeded by adverse weather conditions; for instance, see the works of Rothmeier and Huber [7], Pfeuffer and Dietmayer [8], Hasirlioglu and Riener [9], Chaturvedi et al. [10] among many research papers on this topic. Morrison et al. [11] conducted a user study on the requirements of first responders in terms of communication systems. Although this study focuses on communication infrastructure requirements, nevertheless it points out a need for robust technological solutions that may improve the capabilities of first responders in adverse weather conditions. However, first responders often need to operate in adverse weather conditions, which can degrade both vision and the efficacy of vision-based algorithms. These can include rain, snow, haze, darkness, smoke, dust and others. From a visual computing point of view, these can be divided in two broad categories:

- Conditions which degrade vision without fully obstructing it: such as rain, snow and haze. As objects or people are visible through such conditions both to the naked eye and RGB camera sensors, visual computing algorithms can be used to restore such images and improve visibility.
- Conditions which fully obstruct some or all parts of the field of view: such are total darkness, heavy smoke or dense dust. Such conditions beyond the capabilities of RGB sensors and computer vision algorithms to restore, necessitating other modalities and approaches, such as infrared sensors.

The present survey focuses specifically on the first category, that of conditions that only partially obscure vision, examining in particular the cases of rain, snow and haze. To that end, it presents and categorizes such image restoration methods that can improve clarity of vision in such adverse conditions, both for the responders themselves and for any computer algorithms that they may employ.

Due to the extensive research on the image restoration task, there are numerous research works devoted to the deraining, desnowing and dehazing tasks. While previous surveys have focused on general-purpose image denoising, including deblurring and super-resolution (such as the recently published work by Su et al. [12]), an up-to-date survey of image restoration methods addressing specifically adverse weather conditions and their applicability for disaster response operations, has been missing. To this end, the present work focuses on image restoration methods that could serve the purpose of augmenting the sight of FRs as the means to increase their situational awareness. The augmentation of vision scenarios that we consider regard the existence of adverse weather conditions. That implies that we have focused mostly on architectures that provide accurate results and could be implemented on portable devices that can provide real-time processing. However, non-real-time or near-real-time methods are also taken into consideration, since they may possess several properties or ideas which are desirable in the implementation of new, lightweight models in the future. In our article, we provide an up-to-date survey of the deraining, desnowing and dehazing methods, being employed as important tools for augmenting the sight of FRs and for providing higher-quality input to CV algorithms that are required to make critical inferences.

The key contributions of this survey are as follows:

- We survey the research literature on the deraining, desnowing and dehazing methods that employ DL-based architectures. To the best of our knowledge, this work is the first survey of image restoration methods in adverse conditions for assisting FRs situational awareness.
- We provide a faceted taxonomy of the abovementioned image denoising methods in adverse weather conditions in terms of their technical attributes.
- We compare the existing algorithms in terms of quantitative metrics and processing time in order to decide the appropriateness of each method for the specific task of facilitating the FRs’ vision.

Our work aims to conduct a detailed and comprehensive survey on single-image restoration methods in adverse weather conditions, namely rain, haze and snow. We expect that this study can contribute toward understanding the current trends in the existing methods, their applicability and limitations in an applications level for FRs. Additionally, the challenges that arise from these limitations provide valuable insights for future research directions. The article is structured as follows. In Section 1, we motivate the use of image denoising methods for the removal of rain, haze and snow under the more general context of augmenting the vision capacity of FRs operating in rescue missions. In Section 2, we present the existing datasets for each task. In Section 3, Section 4 and Section 5 we survey the literature of DL-based deraining, desnowing and dehazing methods respectively. For each task, we present a technical taxonomy of the existing literature based on the architecture of the models both for single and multi-images. In Section 6, we present the most common quantitative metrics and the results of the models proposed in the literature in terms of the quantitative metrics and processing time. Last, in Section 7 we summarize the paper.

## 2. Datasets

The availability of datasets for the deraining, desnowing and dehazing tasks is important for the development and cross-evaluation of methods built around these problems. Creating large-scale datasets for training DL models from scratch is not an easy task, therefore, pre-existing publicly available datasets are very important, because they accelerate the development of algorithms and researchers are not required to build their own datasets themselves. In this section we survey the available datasets for the deraining, desnowing and dehazing problems. Table 1, Table 2 and Table 3 summarize the proposed datasets for deraining, desnowing and dehazing, respectively.

### 2.1. Deraining Datasets

A plethora of datasets have become available for enabling the development and evaluation of deraining methods. When designing new deraining methods, a major problem that is faced is the infeasibility of simultaneously capturing pairs of rainy and clean images, thereby imposing difficulties in training supervised models. A notable example refers to the deep end-to-end models, where a pipeline learns to map rainy images fed at the one end to clean images output at the other end. This difficulty has motivated the idea of generating synthetic deraining image datasets. Based on this idea, the designer of the dataset injects noise in the images by means of a synthetic rain streak generation algorithm. Therefore, by knowing where each rain streak is placed, it is easy to obtain a rain streak mask image that can provide a supervisory signal to machine learning (ML) methods. A limitation that is often met when reusing synthetic datasets for training deraining models, is that the injected noise is often not realistic enough for the trained models to be able to generalize well on real-world images with naturally generated rain streaks. Finally, apart from the datasets containing pairs of clean and synthetic rainy images, another class of datasets contain only real-world images for which ground-truth is unknown. Deraining datasets that do not contain ground-truth are more suitable for unsupervised deraining methods (for example, see [13,14,15]), or for semi-supervised deraining methods which can be trained on a combination of a paired dataset and a dataset with no ground-truth (for an example, see [16]).

The Rain*X* datasets (where *X* is an integer value from a fixed set of integers) are some of the most commonly used datasets in the evaluation of deraining algorithms. The Rain12600 dataset is a synthetic rain dataset that was originally contributed by Fu et al. [17]. The dataset contains a total of 14,000 pairs of rainy and clean images of the same scene out of 900 original scenes. To create the dataset, originally 1000 images of scenes were selected from the UCID [18], the BSD [19] dataset and Google image search. For each single image out of the available 1000 ones, 14 rainy images are synthetically generated. Some authors reusing this dataset keep 90% of the available 14,000 14-tuples for training models (amounting to 12,600 images), and keep the rest of the images for testing (amounting to 1400 images). The Rain12000 dataset is another synthetic rain dataset that contains 12,000 images. The dataset was originally introduced by Zhang and Patel [20]. Depending on the rain synthesis occurring in each image, an image is assigned one of three possible labels, namely light-rain, medium-rain and heavy-rain. The Rain1400 dataset is traced back to the publication of Fu et al. [17]. This dataset is a subset of 100 14-tuples from the Rain12600 dataset. The number of examples in this dataset amounts to 10% of the total example images provided by Rain12000. The dataset is commonly referred to as Rain1400, and is a subset of the Rain12600 dataset. The Rain800 dataset was contributed by Zhang et al. [21]. The dataset is split into a training set of 700 synthetic images and a testing set of 100 images. The Rain12 dataset [22] contains 12 synthetic images which are injected with one type of rain streak.

The Test100 and Test1200 datasets were contributed for the purpose of conducting model validation for deraining methods. More specifically, the Test100 dataset was introduced by Zhang et al. [21]. Test100 contains the last 100 examples of the Rain800 dataset. The Test1200 dataset was contributed in the work of Zhang et al. [20]. It contains 1200 images of rainy scenes.

The RainTrainH and RainTrainL datasets make a distinction among scenes with heavy rain and scenes with light rain. The RainTrainH and RainTrainL datasets were contributed in the work by Zhang and Patel [20].

The Rain100H, Rain100L and Rain200H are three of the most commonly used datasets on the deraining problem. Specifically, the Rain100H dataset was contributed in the work of Yang et al. [23]. It is a synthetic rain image dataset, originally composed of samples from the BSD200 dataset [19]. The Rain100L dataset, like the Rain100H dataset, was contributed by Yang et al. [23]. It is a synthetic rainy image dataset that comprises five different orientations of rain streaks. This dataset can be specifically used to test the ability of a deep neural deraining model to learn the regularity of rain streaks. The Rain200H dataset was contributed by Yang et al. [24]. The dataset contains 1800 paired training images and a testing set of 200 paired images.

The Rain in Driving (RID) and Rain in Surveillance (RIS) datasets resemble two use cases of image deraining algorithms, making both of them suitable for validating algorithms whose target application partially or fully coincides with the two use cases. The RID dataset (contributed by Li et al. [25]) is a set of 2495 rainy images extracted from driving videos of a high resolution. The dataset demonstrates the veiling effect of rain streaks on the camera lens. The dataset was captured under real multiple drives and traffic locations. Finally, the dataset contains ground-truth for four objects (namely, car, person, bus and bicycle). The RIS dataset (also contributed by Li et al. [25]) contains a total of 2048 real rainy images from lower-resolution surveillance video cameras. The dataset demonstrates the “rain and mist” scenario where the surveillance cameras introduce a fog-like effect and a mist-like effect caused by rain falling near the cameras.

The DAWN/Rainy dataset (contributed by Kenk and Hassaballah [26]) contains 200 images of outdoor rainy scenes. The dataset can be used for the single image deraining task. It can also be used for an object detection task where the detected targets can be matched directly with object ground-truth, which is available for each image. A limitation of the provided object ground-truth is that the provided ground-truth objects are selective of a small target set of objects.

The NTURain dataset was contributed by Chen et al. [27]. It is a synthetic rain dataset comprising a total of eight video sequences. The frame-count of each video sequence ranges between 200 and 300 frames. A total of four of the videos contain short scenes captured from a panning and unstable camera. The rest of the four videos were captured by a fast moving camera.

The SPA-Data dataset by Wang et al. [28] contains 29,500 rainy or clean image pairs. The data are split into a training set of 28,500 examples and a testing set of 1000 examples. These rainy/clean image pairs are generated from a large dataset of 170 videos of real rain. The videos cover the topics of urban scenes, suburb scenes and outdoor fields.

**Table 1:** Listing of datasets used for the deraining task.

Dataset	Synthetic (S)/Real (R)	Indoor (I)/Outdoor (O)	Pairs	Year
Rain12600 [17]	S	O	14,000	2017
Rain12000 [20]	S	O	12,000	2018
Rain1400 [17]	S	O	1400	2017
Rain800 [21]	R	O	800	2020
Rain12 [22]	S	O	12	2016
Test100 [21]	S	O	100	2020
Test1200 [20]	S	O	1200	2018
RainTrainH [20]	S	O	1800	2018
RainTrainL [20]	S	O	200	2018
Rain100H [23]	S	O	100	2020
Rain100L [23]	S	O	100	2020
Rain200H [24]	S	O	2000	2017
RID [25]	R	O	2495	2019
RIS [25]	R	O	2048	2019
DAWN/Rainy [26]	R	O	200	2020
NTURain [27]	S	O	8 (videos)	2018
SPA-Data [28]	R	O	29,500	2019

### 2.2. Desnowing Datasets

A list of benchmark datasets has been compiled for use in the image desnowing problem. The image desnowing problem has received a limited focus by researchers, in comparison to other problems such as the deraining and dehazing problems. In this section, we describe four important datasets which have been used to assist the development and evaluation of desnowing methods.

The Snow-100K dataset contributed by Liu et al. [29] comprises both synthetic snowy images and realistic images, all downloaded from the Flickr service. In particular, the dataset offers 100,000 synthetic snow images paired with corresponding snow free ground-truth images. The data are split in a training set and a testing set of 50,000 images each. A total of 1329 realistic snowy images are provided along with the synthetic images. The dataset consists of three subsets with small snow particles, medium-size and small-size snow particles, and combined small, medium and large snow particles. Each subset contains around 33,000 images.

The Snow Removal in Realistic Scenario (SRRS) dataset is a dataset that considers the veiling effect of snow, consisting of 15,000 artificially synthesized images and 1000 real-life snow images downloaded from the internet. The popular RESIDE dataset is used to populate the SRRS dataset with images, synthesizing the veiling effect in each one of these images. To provide labeled data of snow particle appearance, various types of snow are synthesized by means of image processing software and labels are assigned to each particle. In terms of a training and validation set, 2500 images are chosen randomly for model training and 1000 images are kept for testing models. The same partition of the images is considered to render the additional case where the veiling effect is absent.

The Comprehensive Snow Dataset (CSD), originally proposed by Chen et al. [30], contains 10,000 synthetic images, borrowed from the well-known RESIDE dataset. A snowflake and snow streak synthesis algorithm is applied to generate synthetic images from the clean images. The variety in the data is controlled in terms of three factors, namely (a) the level of transparency of snow streaks; (b) the size and the location of snow streaks. The goal is to generate realistic looking image samples.

The SITD dataset was contributed in the work of Li et al. [31]. It contains 3000 snowy images, snow-free images and snowflake images. The images were generated from 50 videos that were collected from the web and 100 videos that were recorded by the authors. White snow particles were generated synthetically based on a snow model and were injected in the images. The dataset also promotes the variety of its samples by considering: (a) the density of snowflakes; (b) the shapes of snow particles; (c) the transparency of snowflakes; (d) the time of the day at which samples were captured; (e) the scene that was captured.

**Table 2:** Listing of datasets used for the desnowing task.

Dataset	Synthetic (S)/Real (R)	Indoor (I)/Outdoor (O)	Pairs	Year
Snow-100K [29]	S & R	O	100,000+	2018
SRRS [32]	S & R	I & O	16,000	2020
CSD [30]	S	I & O	10,000	2021
SITD [31]	S	O	3000	2019

### 2.3. Dehazing Datasets

A great amount of datasets focusing on the dehazing task has been found in the literature. Most of them are proposed for training supervised learning models and therefore contain pairs of hazy and haze-free images. Since it is impossible to capture the exact same image with and without haze simultaneously, researchers have approached this problem in three different ways. The first one is using datasets in which haze is synthetic and has been added to clear images based on mathematical models; the second one is using datasets in which haze is generated by a professional haze machine, and the third and most recent one is using datasets where both hazy and clear images are real, but are not captured at the same time. In this section, we describe the most popular datasets used for the dehazing task.

Tarel et al. [33] released the first synthetic dataset for single image dehazing, named Foggy Road Image DAtabase (FRIDA). It consists of 72 pairs of synthetic images with and without fog, captured from virtual urban road scene using the SiVIC™ software. For each one of the 18 clear images, four foggy ones were generated synthetically. In order to generate the foggy images, four different types of synthetic fog were used. The FRIDA2 dataset was introduced by Tarel et al. [34]. It consists of 264 pairs of synthetic images with and without fog, captured from virtual diverse road scenes, where for each clear image four synthetic foggy ones were generated. The synthetic images were generated in the same way as in FRIDA.

Some years later, El et al. [35] released the CHIC dataset. It is a benchmark dataset created using two indoor scenes in a controlled environment that were captured both in hazy and clear conditions. In order to create haze, a haze machine was used and produced nine different levels of haze density. The clear images and their corresponding nine hazy images are also accompanied by some known parameters like the distance between each object and the camera, the local scene depth, etc.

One of the most widely used datasets for single image dehazing is the Realistic Single Image Dehazing (RESIDE) dataset [36]. RESIDE is a benchmark dataset that consists of two versions: the Standard and the extended (RESIDE-β) one. The standard version includes: (a) an Indoor Training Set (ITS) which contains over 10,000 pairs of hazy and clear indoor images, where for each clear image 10 synthetic hazy ones were generated; (b) a Synthetic Objective Testing Set (SOTS), which contains 500 pairs of hazy and clear indoor images produced in the same way as in ITS; (c) a Hybrid Subjective Testing Set (HSTS), which contains: 10 pairs of real, clean, outdoor images and the generated hazy ones and 10 real, outdoor hazy images. The RESIDE-β includes: (a) an Outdoor Training Set (OTS) which contains over 70,000 pairs of hazy and clear outdoor images, where for each clear image 35 synthetic hazy ones were generated; (b) a Real-world Task-driven Testing Set (RTTS), which contains over 4000 real hazy images annotated with object categories and bounding boxes.

Other benchmark datasets include the D-HAZY [37], I-HAZE [38], O-HAZE [39], DENSE-HAZE [40] and NH-HAZE [41] datasets proposed by Ancuti et al. The D-HAZY [37] dataset contains over 1400 pairs of hazy and clear indoor images. It is built on the Middelbury [42] and NYU Depth [43] datasets, which are datasets that provide clear images and their corresponding depth information. The hazy images were synthesized by taking into consideration depth information and by using the physics-based model presented in Section 6.3.5. The I-HAZE [38] dataset contains 35 pairs of hazy and clear indoor images. Here, both hazy and clear images are real and captured under the same illumination conditions. Haze is produced artificially by a professional haze machine and all the images were taken in a controlled environment. The O-HAZE [39] and the DENSE-HAZE [40] datasets contain pairs of hazy and clear outdoor images. DENSE-HAZE [40], which contains 33 pairs of images can be considered as the extension of the O-HAZE [39] dataset, which contains 45 pairs of images, in the way that it contains a much denser and challenging haze than O-HAZE. The images in both datasets were captured in the same way as in I-HAZE. The NH-HAZE [41] dataset contains 55 pairs of hazy and clear outdoor images, captured in the same way as in I-HAZE, O-HAZE and DENSE-HAZE datasets, but here the haze in the images is non-homogeneous.

The Haze Realistic Dataset (HAZERD) [44] is a benchmark dataset that contains 14 clear, outdoor images accompanied by their corresponding depth maps estimated by fusing structure from motion and LIDAR. Using the depth map of a clear image, they synthesized five hazed ones, where each one of them has different levels of haze density.

Zhao et al. [45] recently released the BEnchmark Dataset for Dehazing Evaluation (BeDDE). BeDDE is a real-world outdoor benchmark dataset where both hazy and clear images are real and consists of two versions: the standard BeDDE and the EXtension of the BeDDE (exBeDDE). The standard BeDDE contains 208 pairs of hazy and clear images, collected from 23 provincial capital cities in China. One clear and more than one hazy images were captured from the same place for each city. The images are accompanied by manually labeled masks that provide the regions of interest and the hazy images are also manually sorted into three categories on the basis of their haze density levels (“light”, “medium” and “heavy”). The exBeDDE contains 167 hazy images from 12 cities selected from the BeDDE dataset accompanied by 1670 dehazed images. For each hazy image 10 representative models were selected for producing the corresponding dehazed images, and for each dehazed result a subjective score from people is provided for assessing the performance of the dehazing evaluation metrics.

Zheng et al. [46] introduced the 4KID dataset, a 4K resolution (3840×2160) dataset, which consists of 10,000 pairs of hazy and clear images extracted from 100 videos. The haze was synthesized using the physics-based model presented in Section 6.3.5 and a translation module that translates the hazy images from the synthetic to the real domain.

Recently, the first REal-world VIdeo DEhazing (REVIDE) dataset [47] was released. It has given the opportunity to further research the video dehazing problem. This benchmark dataset contains 48 pairs of hazy and haze-free videos from scenes with 4 different styles. The haze is produced artificially by a professional haze machine and all the videos were taken in a controlled environment.

**Table 3:** Listing of datasets used for the dehazing task.

Dataset	Synthetic (S)/Real (R) /Generated (G)	Indoor (I)/Outdoor (O)	Pairs	Year
FRIDA [33]	S	O	72	2010
FRIDA2 [34]	S	O	264	2012
CHIC [35]	G	I	18	2016
RESIDE [36]	S & R	I & O	10,000+	2018
D-HAZY [37]	S	I	1400+	2016
I-HAZE [38]	G	I	35	2018
O-HAZE [39]	G	O	45	2018
DENSE-HAZE [40]	G	O	35	2019
NH-HAZE [41]	G	O	55	2020
HazeRD [44]	S	O	70	2017
BeDDE [45]	R	O	200+	2020
4KID [46]	S	O	10,000	2021
REVIDE [47]	G	I	40+ (videos)	2021

Although there exist a satisfactory number of publicly available datasets for the deraining, desnowing and dehazing problems, unfortunately none of them contain scenes representative of the FRs’ working environment. Additionally, in most of the paired datasets the noise is either synthetic or generated artificially using a machine. This leads to the fact that most of the models proposed in the literature suffer from the domain shift problem and they are not able to perform well in real-world scenarios.

## 3. A Review of the Deraining Literature

Rain is one of the most common bad weather conditions. When capturing an image in a rainy scene, the blurring effect and haziness are noticeable phenomena causing generally a degradation of the visual quality [48]. This degradation can affect FRs’ visual perception as well as the performance of any CV tool employed to help their operations in outdoor scenes. In the last decades, rain removal, which is referred to as deraining, is a task that has attracted a lot of interest in the research community. In this Section, we survey the recent bibliography on the deraining problem. Most of the presented methods target the single image deraining problem, in which the goal is to restore a clean image from an input image which is corrupted by rain streaks. Other methods target the deraining problem from the standpoint of other modalities, such as image sequences (video) or stereo images.

### 3.1. A Taxonomy of the DL-Based Single Image Deraining Methods

In this Section, we provide a taxonomy of the surveyed deraining methods in terms of the methodological attributes that may characterize a particular deraining algorithm. To provide a more compact representation of the collected works on the deraining problem, Table 4 directly summarizes the methods they present in keywords, and further categorize some of their common attributes (for instance, their general model idea or their use of an underlying imaging equation).

Previous surveys on the subject [49,50,51,52,53] all provide a similar categorization of the surveyed methods. In this survey paper, we particularly highlight the attributes of more recent methods; that is, methods that were published in the year 2020 onward. By inspecting the reviews compactly being presented in Table 4, we came up with a set of seven methodological attributes which characterize the surveyed methods. Each method presented in this Section may be tied with at least one of the suggested attributes. For example, a method may simultaneously employ a generative adversarial network-like architecture and harness this architecture by means of an attention mechanism.

#### 3.1.1. CNN-Based Deraining Methods

Deraining networks based on deep convolutional neural networks (CNNs) are networks which are mainly composed by convolutions, pooling layers, dropout layers, or other layers which are common in vanilla deep CNNs. These plain models usually do not contain more sophisticated structures such as attention layers, multi-scale analysis constructions, etc.

Fu et al. [17] propose the DetailNet method, which uses a CNN model leveraging prior domain knowledge from the high-frequency content of training images. Then, by doing so the model can learn the structure of rain streaks from training data. The authors empirically observe that the learnt model can also generalize well on real data when trained only on only synthetic data. Fu et al. [48] propose the DerainNet model. The model employs a deep CNN and uses the high-pass frequency layer computed from input images. As in the model presented by Fu et al. [17], this model can also generalize well on real-world rain images with the underlying model being trained on synthetic data. Yang et al. [54] injected a hierarchical wavelet transform into a recurrent process that considers the low-frequency component computed from an input image. The learnt network can adapt to rain streaks of a larger size while being trained on image examples with rain streaks of only one size.

Coarse-to-fine or local-to-global strategies are also common in the general deraining literature. Fan et al. [55] propose the ResGuideNetwork model. This model can progressively produce high quality deraining results, and it has a small set of parameters. It implements a novel residual-guide feature fusion network. A cascaded network is proposed and residuals are adopted from shallower blocks to provide data to blocks that are at deeper positions in the network. A coarse-to-fine estimation of negative residuals is also realized in the overall model. Wang et al. [28] propose an image deraining method that is based on human supervision and temporal priors in order to derain images. It furthermore entails a novel SPatial Attetive Network (SPANet) to remove rain streaks with a local-to-global removal strategy.

Li et al. [56] proposes the NLEDN model, an end-to-end encoder-decoder network with pooling layers, suitable for efficiently learning increasingly abstract feature representations. The encoder-decoder design implements non-locally enhanced dense blocks. These blocks are useful for learning features of a hierarchical structure, and for learning long-range feature dependencies. Pan et al. [57] propose the dual CNN (DualCNN) for super-resolution, edge-preserving filtering, deraining and dehazing. The network consists of two parallel branches, recovering the structures and details end-to-end. The DualCNN can be integrated with common CNN models. Huang and Zhang [58] propose a deraining network employing the Dynamic Multi-domain Translation (DMT) module. The parameters of the DMT-Net are estimated end-to-end.

Zhang and Patel [20] propose the DID-MDN multi-stream densely connected CNN for joint rain density estimation and deraining. The network can learn the density of rain from training images and can use features computed at different scales. The reader can also be pointed to the multi-scale methods reviewed in Section 3.1.5.

Cho et al. [59] proposes a deraining network based on a memory network that explicitly helps to capture long-term rain streak information. The memory network is tied with an encoder-decoder architecture, similarly with the works by Li et al. [56]; Pan et al. [57]; and, Li et al. [25]. The features that are extracted from the encoder are read and updated within the memory network.

Guo et al. [60] views single image deraining as a predictive filtering problem. Via their method, they predict proper kernels via a deep neural network that filters out pixels in rainy images. The model addresses so-called residual rain traces, multi-scale and diverse rain patterns by achieving deraining efficiency.

Zheng et al. [61] propose the Segmentation-aware Progressive Network (SAPNet) based on contrastive learning. First, a lightweight network comprising Progressive Dilated Units (PDUs) is formed. The SAPNet model also implements an Unsupervised Background Segmentation (UBS) network, which can preserve semantic information and improve the generalization ability of the network. Learning the model occurs by optimizing the perceptual contrastive and perceptual image similarity loss functions.

Li et al. [25] proposes a novel multi-task learning, end-to-end architecture. The application of multi-task learning in this model is similar to that applied by Yang et al. [23]. The decomposition network splits rain images into two layers: the clean background image layer, and the rain-streak layer. During the training phase, the authors further employ a composition structure to reproduce the input by the separated clean image and rain information for improving the quality of the decomposition. Jiang et al. [62] propose the Progressive Coupled Network (PCNet) to separate rain streaks from the useful background regions of rainy images. The integrated Coupled Representation Model (CRM) learns the joint features and blending correlations. A low-memory requirement for the method is made possible by the incorporation of depth-wise separable convolutions and a U-shaped neural network structure.

#### 3.1.2. Different Learning Schemes for Deraining

In this Section, we mention a few works on the single image deraining problem that are based on classic ML schemes that are common in the general literature of ML. The target learning schemes met here are multi-task learning, self-supervised learning and semi-supervised learning.

Yang et al. [23] developed the JORDER method. The method uses multi-task learning to jointly learn a binary rain map, and finally estimates the rain streak layers and the clean background. The authors introduced the contextual dilated network, able to exploit regional contextual information. A recurrent process progressively removing rain streaks can handle rain streak variability.

The Few Shot Self-Supervised Image Deraining (FLUID) method by Rai et al. [63] proposes a self-supervised technique for single image deraining. As the authors mention, self-supervised approaches rely on two assumptions: (a) the distribution of noise or rain is uniform, and (b) the value of a noisy or rainy pixel is independent of its neighbors. To overcome these problems, the authors of FLUID hypothesize a network trained with minimal supervision to compute the probabilities of pixel rain-class membership.

Wei et al. [16] propose a semi-supervised learning approach where singleton rainy image examples are used, without their paired corresponding clean images. The network learns to use the regularities of the data in the unlabeled examples by first bootstrapping a model given the examples with labelled rain information.

#### 3.1.3. Generative Models for Deraining

The adversarial learning framework developed by Goodfellow and colleagues [64] targets to learn a generative model of the underlying data distribution by being given a finite training dataset of labeled examples. The framework learns a generator function *G* (usually taking the form of a multilayer perceptron) from which we can sample examples, and a discriminating function *D* that computes the probability of an example being a real example drawn from the training set. To learn both functions given the training data, the framework alternates the optimization of the parameters of the two functions (the generator and the discriminator function), rendering a minimax 2-player optimization game. This game is represented by a value function, which is in turn a function of the parameters of the generating and the discriminator functions. The framework attempts to sequentially alternate among minimizing the value function as a function of the generator’s parameters, and then maximize it in terms of the parameters of the discriminator function.

Four of the surveyed research papers and articles that we collected in this survey, are based on the generative adversarial learning scheme by Goodfellow et al. [64]. Otherwise, an altered adversarial learning scheme is used. Here, we briefly discuss how they work.

Qian et al. [65] present an altered adversarial learning scheme for single image deraining. The scheme employs an altered generative adversarial network formalism, in which the generator function uses a contextual autoencoder. The autoencoder requires an input image, and the attentive-recurrent module computes an attention map for the input image. The multi-scale loss and the perceptual loss, are employed for the autoencoder to be able to extract contextual information off from different scales (see also multi-scale methods in Section 3.1.5). The discriminator function enforces global and local image content consistency. Global-to-local and coarse-to-fine strategies are also discussed in Section 3.1.1.

**Figure 1:**
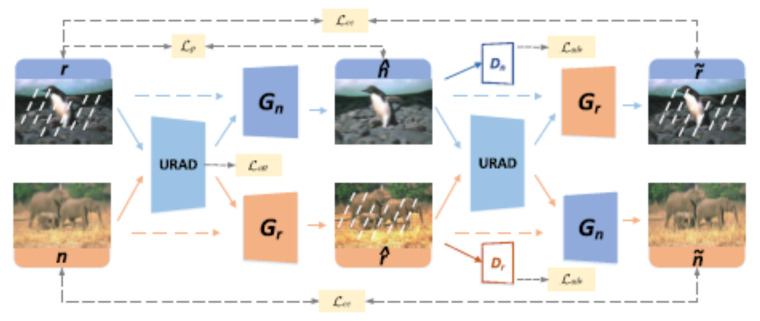
Schematic of the DerainCycleGAN method by Wei et al. [66]. The model follows the CycleGAN regime and employs URAD (Unsupervised Rain Attentive Detector) modules to attend to rain information and guide intermediate data projections.

Guo et al. [15] propose the DerainAttentionGAN model for single image deraining. The generative adversarial model scheme used in this framework contains two generator functions *G* and *F* and two corresponding discriminator functions $D_{clean}$ and $D_{rain}$. The cycle consistency loss involves these functions and affects the parameters of the network under the condition that F(G(rain))≈rain is obeyed approximately. The generator function *G* is linked with three subnetworks: (a) the parameter sharing feature extractor $G_{F}$; (b) the attention network $G_{A}$; and, (c) the transformation network $G_{T}$. Wei et al. [66] propose the DerainCycleGAN model (for a graphical representation of this model, see Figure 1), providing an adversarial learning-based methodology for single image deraining. The model follows the CycleGAN formalism by Zhou et al. [67], and employs the unsupervised rain attentive detector (URAD) to attend to rain information in both rain and rain-free image inputs.

Yang et al. [68] propose a generative adversarial learning scheme, typically including a generator and a discriminator function. The authors integrate capsule units in both the generator and the discriminator functions in order to further improve their discriminant abilities in generatively identifying the characteristic structure of rain streaks in target images. The method is optimized by iteratively minimizing the loss functor in terms of the generator and the discriminator functions. Moreover, a Rain Component Aware (RCA) loss is introduced to minimize the differences between the synthesized rain component map and the ground-truth by forwarding them through the pretrained network (termed the RCA network).

Jin et al. [13] proposed the UD-GAN method, which relies on an altered adversarial learning formulation. This formulation includes a rain generation function that takes into account the variation of style and color.

The RainGAN model [69] is trained to decompose a rainy image into a clean image component and a latent 2D matrix encoding raindrop structures in the rainy image. It can also reverse this mapping and map a clean image and a raindrop latent code into an image with raindrops. This mapping is defined by the implementation of domain-invariant residual blocks. HeavyRainStorer [70] is a method specifically designed to handle images contaminated by heavy rain, which can cause the veiling effect. The proposed method is a network consisting of two processing stages. In the first stage, a physics-based model is applied to remove rain streaks from an input image, the transmission and the atmospheric light. In the second stage, a depth-guided Generative Adversarial Network (GAN) recovers the background details and corrects artifacts caused by the first processing stage.

The ID-CGAN (Image Deraining Conditional Generative Adversarial Network) [21] method considers quantitative, visual and discriminative performance in a common objective function mimicking the form of the conditional GAN loss functions.

#### 3.1.4. Attention Mechanisms for Deraining

In DL, neural attentive mechanisms are mathematical constructs which are integrated in the DL model and are applied on training data, allowing for the network model to attend to some interesting portions of the data while adaptively neglecting other data that are probably uninteresting to the model and most probably to a human interpreting the model. A model learns to adapt an attention construct to the data by means of model optimization. In this paragraph, we briefly mention the attentive mechanisms employed by the deraining methods which are reviewed in our survey.

Jiang et al. [71] propose a neural network that decomposes images with rain streaks into multiple rain-specific layers. Each consequent layer is individually analyzed in order to identify rain streak structures. Improved non-local blocks are designed to exploit the self-similarity in rain information by learning spatial feature correlations, while simultaneously reducing computation time. A mixed attention mechanism is applied to guide the fusion of rain layers.

Hu et al. [72] present the DAF-Net model. The model is trained to learn depth-attentional features from the training image set. At first, the input image is forwarded through a deep neural network that calculates the depth map of the input image. Then the integrated attention mechanism is applied to the calculated depth map and attention weights are, in turn, computed. The latter weights indicate the local and more distant structure in depicted rain streaks.

The APAN method by Wang et al. [73] employs an attention module that operates at an across-scale manner to capture long-range feature correspondences from computed multi-scale features. The dual attention residual group network (DARGNet) by Zhang et al. [74] proposes a single image deraining model for increased performance on the task. The framework comprises two attention mechanisms: a spatial attention mechanism and a channel attention one. The spatial attention mechanism extracts multi-scale features that can capture the variability of both shape and size in rain streaks. In turn, the channel attention mechanism calculates signal dependencies that exist among different channels. We can draw a similarity with the method of Jiang et al. [71] in the computation of cross-channel signal dependencies. Wei et al. [75] proposed a hybrid neural network with an integrated robust attention mechanism, termed the Robust Attention Deraining Network (RadNet). The authors designed a lightweight network with a universal attention mechanism for rain removal at a coarse level, and then proposed a deep neural network design with multi-scale blocks for finer rain removal.

The recent method by Zhou et al. [76] proposes a task adaptive attention module to enable the neural network to restore images with multiple degradation factors. Moreover, the model also employs a task channel attention module and a task operation attention module.

Lin et al. [77] proposed an Efficient Channel Attention (ECA) module that can extract global information and detailed information adaptively, resulting in a network module that is extremely lightweight.

**Figure 2:**
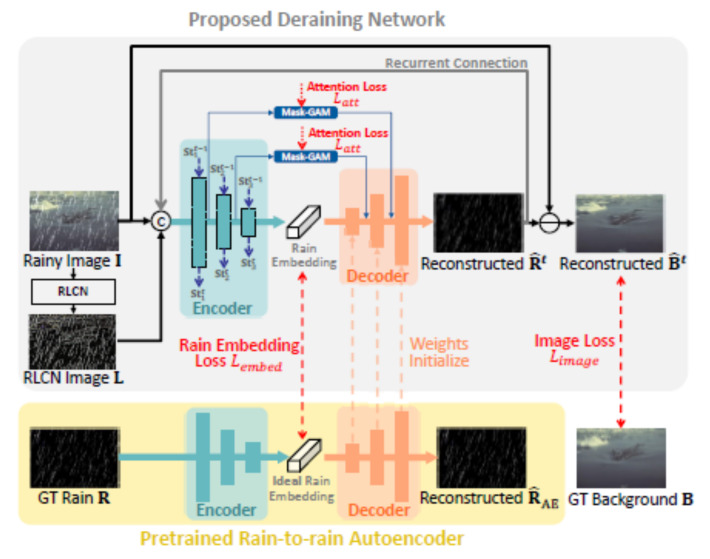
Schematic of the SIR method by Li et al. [78]. The model employs an autoencoder for single image deraining, regularization loss functions and deterministically generated features that encourage the autoencoder to attend to rain streak features.

Li et al. [78] proposed an encoder-decoder-based model employing the rain embedding loss. This loss is used to supervise the encoder. The Rectified Local Contrast Normalization (RLCN) is used to extract candidate rain pixels. The authors also propose to use a Long Short-Term Memory (LSTM) network for recurrent deraining and fine-grained encoder feature refinement at different scales. Figure 2 shows the schematic of the method.

#### 3.1.5. Multi-Scale Based Deraining Methods

Extracting information from multiple scales off from training data or their intermediate representations may reveal complimentary structures which may be usable in subsequent neural processing. In this Section, we review the multi-scale data analysis strategies followed by the single image deraining literature.

Wei et al. [75] introduces the RadNet model. The model comprises a Robust Attention Module (RAM), and a universal attention mechanism for rain removal at a coarse level. Moreover, a Deep Refining Module (DRM) comprising multi-scale blocks allows for fine rain removal.

The LFDN network by Zhang et al. [79] employs the Multi-scale Hierarchical Fusion Module (MSHF). The input to this module is an initial feature map. Intermediate features computed from the initial feature map are extracted using downblock modules. The MSPFN method by Jiang et al. [80] employs a multi-scale collaborative representation for rain streaks. This framework is unified and can model rain streaks in terms of their scale and their hierarchical deep features.

Fu et al. [81] exploits underlying complimentary information not only across multiple scales but also between channels. The network is designed to transmit the inter-level and inter-scale features in the neural processing pipeline. The Dual Attention Residual Group Network (DARGNet) by Zhang et al. [74] integrates a dual attention model, which comprises spatial attention and channel attention that can integrate scale and channel information.

The Dense Feature Pyramid Grids Network (DFPGN) by Wang et al. [82] computes multi-scale features from an input image and then shares these features through several pathways across layers.

Yamamichi and Han [83] introduces a novel, multi-scale, residual-aggregation deraining network called MRADN. A residual backbone extracts fine and detail context in an initial scale. A multi-scale analysis module augments feature learning from a semantic context representation.

Zheng et al. [61] proposed the Segmentation-Aware Progressive Network (SAPNet) method. The model is lightweight and repetitively employs Progressive Dilated Units (PDUs). The PDU is reported to crucially expand the receptive field and allow for a multi-scale analysis of rain streaks.

**Figure 3:**
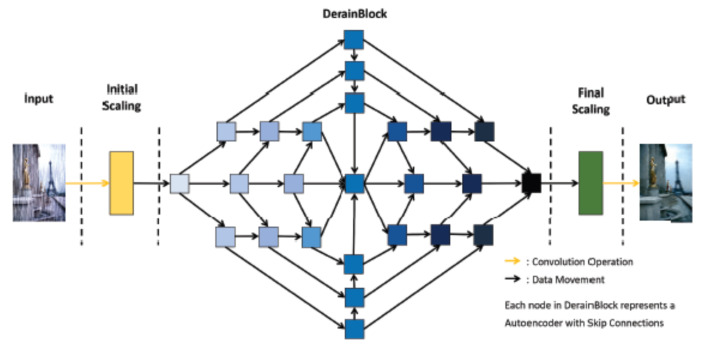
Schematic of the SphinxNet method by Jasuja et al. [84]. Encoder and decoder modules are arranged hierarchically to allow for multi-scale feature encoding and decoding.

Jasuga et al. [84] developed the SphinxNet deraining neural network (see Figure 3). The model can be described in terms of three computation phases: (a) data scaling; (b) the DerainBlock phase, which entails the feedforward pass of initial scaling data across hierarchically placed local encoder and decoder networks; and, (c) final scaling. After the last step, a derained output image is reported.

#### 3.1.6. Recurrent Representations for Deraining

Recurrent representations in DL are deep neural networks which are implemented as a recursive function that is, in turn, a function of a set of parameters. The next term in the recursive function depends on the previous term or on more than one of the previous terms, assuming a set of model parameters.

**Figure 4:**
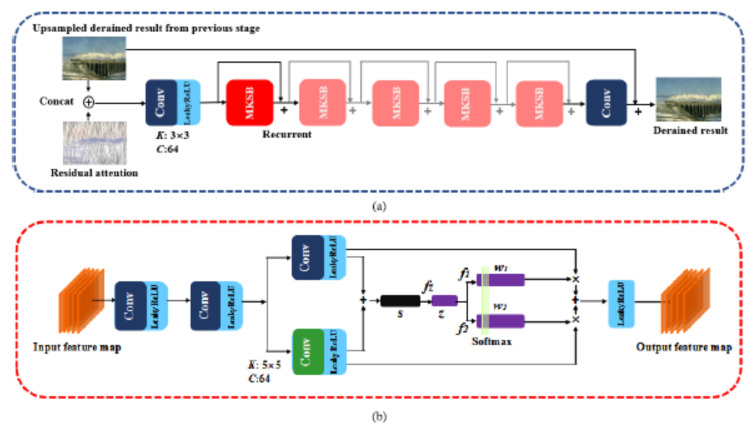
Schematic of the method by Zhang et al. [85]. The (**a**) shows the MSKSN model, and (**b**) shows the MKSB module.

Zheng et al. [85] employ a model that is lightweight and contains recurrent layers to sequentially remove rain at each level of a pyramid. Figure 4 shows the schematic of the method. Fu et al. [86] proposes the lightweight pyramid network (LPNet) for the task of single image deraining. It leverages domain-specific knowledge and Gaussian-Laplacian pyramid decomposition in order to simplify learning globally and at each pyramid level, respectively. Recursive and residual network structures are employed to assemble the LPNet, maintaining a low count of network parameters and state-of-the-art performance. Li et al. [87] introduces the RESCAN model. Contextual dilated networks are employed by this model to iteratively (or progressively) remove rain streaks. Squeeze-and-excitation blocks are used to estimate the strength weight of various rain streak layers before they are linearly combined to approximate the rain-free image. A recurrent neural network links features generated at the different stages of computation.

The method presented by Su et al. [88] proposes a novel, unified and recurrent convolutional residual network. The model employs the so-called *R*-blocks in convolutional residual networks. Non-local Channel Aggregation (NCA) simultaneously captures long-range spatial and channel correlations. In the popular PReNet model introduced by Ren et al. [89], a recurrent layer exploits the correspondence of deep features computed at different stages. The recursive evaluation of the ResNet model occurring at a single neural processing stage can affect the count of network parameters at the expense of negligible image deraining quality.

Lin et al. [90] uses parallel recurrent subnetworks to distribute the load of identifying rain streaks at certain ranges of streak size.

#### 3.1.7. Data Fusion Strategies for Deraining

The strategies of fusing data in deep neural networks entail the extraction and/or combination of data or information from different sources to different targets. In this Section, we describe the fusion strategies used in the single image deraining bibliography.

The ResGuideNet method by Fan et al. [55] fuses feature activations computed in a layer-by-layer fashion. Similarly with the ResGuideNet model, the DFPGN model proposed by Wang et al. [82] fuses intermediate feature representations at neural processing layers by means of dense connection blocks.

The method by Zhang et al. [91] includes the SFNet and ViewNet neural submodules. SFNet computes neural activations from derained images and their scene segmentations, and concatenates the latent vector representations from both modalities before the concatenated vector is fed to a deep CNN. The VFNet fuses the derained image representation and the scene-segmentation representation obtained from the initial left and right stereo channels. Then, an encoder-decoder network reconstructs the left and right derained images. The Lightweight Fusion Distillation Network (LFDN) by Zhang et al. [79] employs a multi-scale, hierarchical fusion scheme (termed MSHF). The model can encode images with rain and blur artifacts. It also regulates the parameter count of the resulting neural model.

Wang et al. [92] proposes recurrent scale-guide networks for single image deraining. The method goes beyond the case of a monolithic single-stage deraining neural network model; it introduces a recurrent network framework and employs a Long Short-term Memory (LSTM) network to join link neural processing stages simultaneously. Cai et al. [93] propose the dual recursive network (DRN). The method is empirically shown to be fast at image deraining. The increased running time speed is mainly attributed to the very low number of model parameters, and the variable recurrent count that is a model hyperparameter. Besides the low model parameter count, the quality of its output is comparable or better than state-of-the-art approaches (PSNR and SSIM measures are considered by the authors). The DRN method utilizes convolutional feature maps, and a recursive residual network which consists of two residual blocks. The residual blocks are evaluated recurrently, allowing for the model to iteratively refine the deraining transformation. The method resembles other published techniques which progressively refine their output (for instance, see the MSPFN method [80]).

### 3.2. Multi-Image Deraining

Zhang et al. [91] and Kim et al. [94] present two methods that receive as input stereo images. The rest of the methods in this Section, including the work of Kim et al. [94], are video deraining methods.

#### 3.2.1. Stereo-Based Methods for Deraining

Zhang et al. [91] propose the Paired Rain Removal Network (PRRNet). The model comprises a Semantic-Aware Deraining Module (SADM) which can simultaneously perform semantic segmentation and deraining. The Semantic Fusion Network (SFNet) and the View-Fusion Network (VFNet) both fuse semantic information and information obtained from multiple views. Additionally, the Enhanced Paired Rain Removal Network (EPRRNet) uses a prior that describes the semantic information in rain streaks in order to remove rain streaks. A coarse deraining network first reduces the rain streaks on the input images, and then a semantic segmentation network extracts semantic features from the coarse derained image. A better deraining result is obtained by another network that fuses the semantic and multi-view information.

Kim et al. [94] proposes a method for video deraining that makes use of temporal correlations in the sequence of video frames, and uses a low-rank matrix completion method to remove rain streaks in a video sequence. Video deraining is achieved as a sequence of steps: at first, a rain pixel probability map is computed by frame subtraction; secondly, the rain probability map is analyzed in terms of sparse basis vectors; third, the obtained basis vectors are classified into vectors referring to rain streaks or vectors pertaining to outlying information. A low-rank matrix completion technique is then applied to remove rain streaks.

#### 3.2.2. Video-Based Methods for Deraining

Xue et al. [95] propose a real-time method for video deraining. A fast attentive deformable alignment module and a spatial-temporal reconstruction module are used. Moreover, deformable convolution based on the channel attention mechanism is employed to maintain frame continuity. Neural architecture search is also employed to discover an effective architecture for temporal information aggregation. Zhang et al. [96] propose the Enhanced Spatio-Temporal Interaction Network (ESTINet). This model is optimized for better video deraining quality and for data processing speed. Deep residual networks and convolutional LSTM models are employed to capture spatial features and temporal correlations in successive frames. Yan et al. [97] employ a self-alignment network with transmission-depth consistency. The method uses deformable convolution layers in an encoder function for feature-level frame alignment. The temporal relationships among frames are considered in order to make a prediction for the current frame. The model can also handle the accumulation of rain to resolve the ambiguity between the depth and water-droplet density. The network estimates the depth from consecutive rain-accumulation-removal outputs and calculates the transmission map using a physics-based model.

The ADN method by Yang and Lu [98] extracts multi-scale features from input shallow features. The extracted hierarchical, multi-scale features are then concatenated together and fed into a rainy map generator that estimates the rain layer.

The method by Wang et al. [99] proposes the Recurrent Multi-level Residual Global Attention Network (RMRGN) to gradually utilize global attention information and image details to remove rain streaks progressively.

Deng et al. [100] propose the RoDerain network. The model employs the so-called rotation operator to remove the rain streaks in natural and stochastic scenes. The alternating direction method of multipliers is used to optimize the model.

Kulkarni et al. [101] proposes the Progressive Subtractive Recurrent Lightweight Network (PSRLN) for video deraining. The Multi-kernel Feature Sharing Residual Block (MKSRB) learns to capture the structure in rain streaks of a varying size. This allows for the removal of rain streaks through iterative subtraction. Features that are generated by the MKSRB are merged with output generated at previous frames in a recurrent fashion to maintain temporal consistency. The Multi-scale Multi-Receptive Difference Block (MMRDB) performs feature subtraction as the means to avoid detail loss and extract HF information. Yue et al. [102] present a semi-supervised video deraining method, in which a dynamical rain generator is employed to fit the rain layer. The dynamical generator consists of one emission model and one transition model. These both help encode the spatial appearance and temporal dynamics of rain streaks. Different prior formats are designed for the labeled synthetic and unlabeled real data so as to fully exploit their underlying common knowledge. An expectation-maximization algorithm is developed to learn the model.

Yang et al. [103] proposes a two-stage recurrent network with dual-level flow regularization. At a first stage, the architecture extracts motion information from the initially estimated rain-free frame, and motion modeling at a second stage. Furthermore, to maintain motion coherence between frames, dual-level flow-based regularization is proposed. This regularization occurs at a coarse flow level and at a fine-pixel level. Wang et al. [99] and Kulkarni et al. [101] also introduce recurrent processing in the models they propose.

**Table 4:** Listing of surveyed research papers.

Category	Method	Model	Short Description	Year
CNN- based	DetailNet [17]	ACM	reduces mapping range; promotes HF details	2017
	Residual-guide [55]	ACM	cascaded; residuals; coarse-to-fine	2018
	NLEDN [56]	ACM	end-to-end, non-locally-enhanced; spatial correlation	2018
	DID-MDN [20]	ACM	density-aware multi-stream densely connected CNN	2018
	DualCNN [57]	ACM	estimation of structures and details	2018
	Scale-free [54]	HRMLL	wavelet analysis	2019
	DMTNet [58]	ACM	symmetry reduces complexity; multidomain translation	2021
	UC-PFilt [60]	ACM	predictive kernels; removes residual rain traces	2022
	SAPNet [61]	N/A	PDUs; unsupervised background segmentation; perceptual loss	2022
	DDC [25]	SBM	decomposition and composition network; rain mask	2019
	DerainNet [48]	ACM	non-linear rainy-to-clear mapping	2017
	PCNet [62]	MRSL	learns joint features of rainy and clear content	2021
	Spatial Attention [28]	ACM	human supervision; global-to-local attention	2019
	memory encoder–decoder [59]	ACM	encoder–decoder architecture with memory	2022
Attention	APAN [73]	ACM	multi-scale pyramid representation; attention	2021
	IADN [71]	ACM	self-similarity of rain; mixed attention mechanism; fusion	2020
	DECAN [77]	ACM	detail-guided channel attention module identifies low-level features; background repair network	2021
	DAF-Net [72]	DRM	end-to-end model; depth-attentional features learning	2019
	SIR [78]	ACM	encoder–decoder embedding; layered LSTM	2022
	RadNet [75]	ACM/ Raindrop	restores raindrops and rainstreaks; handles single-type, superimposed-type or blended-type data	2021
	DARGNet [74]	HRM	dual-attention (spatial and channel)	2021
	task-adaptive attention [76]	N/A	task-adaptive, task-channel, task-operation attention	2022
Generative models	DerainAttentionGAN [15]	ACM	uses Cycle-GAN; attention	2022
	DerainCycleGAN [66]	ACM	CycleGAN transfer learning; unsupervised attention	2021
	RCA-cGAN [68]	ACM	rain streak characteristics; integration with cGAN	2022
	RainGAN [69]	Raindrop	raindrop removal as many-to-one translation	2022
	UD-GAN [13]	ACM	GAN; self-supervised constraints from intrinsic statistics	2019
	HeavyRainStorer [70]	HRM	2-stage network; physics-based backbone; depth-guided GAN	2019
	ID-CGAN [21]	ACM	conditional GAN with additional constraint	2020
	AttGAN [65]	Raindrop	attentive GAN; learns rain structure	2018
Multi-scale based	MSPFN [80]	N/A	streak correlations; multi-scale progressive fusion	2020
	MRADN [83]	ACM	multi-scale residual aggregation; multi-scale context aggregation; multi-resolution feature extraction	2021
	LFDN [79]	N/A	encoder–decoder architecture; encoder with multi-scale analysis; decoder with feature distillation; module fusion	2021
	SphinxNet [84]	N/A	AEs for maximum spatial awareness; convolutional layers; skip concatenation connections	2021
	DFPGN [82]	ACM	cross-scale information merge; cross-layer feature fusion	2021
	GAGN [81]	ACM	context-wise; multi-scale analysis	2022
	UMRL [104]	ACM	UMRL network learns rain content at different scales	2019
Different learning schemes	JORDER [23]	HRM	multi-task learning; priors on equation parameters	2020
	FLUID [63]	N/A	few-shot; self-supervised; inpainting	2022
	Semi-supervised CNN [16]	ACM	adapts to unpaired data by training on paired data	2019
Recurrent	PReNet [89]	ACM	repeated ResNet; recursive; multi-scale info extraction	2019
	recurrent residual multi-scale [85]	MRSL	residual multi-scale pyramid; coarse-to-fine progressive rain removal; attention map; multi-scale kernel selection	2022
	Scale-aware [90]	HRM	multiple subnetworks handle range of rain characteristics	2017
	RESCAN [87]	Equation (A8)	contextual dilated network; squeeze-and-excitation block	2018
	Pyramid Derain [86]	ACM	Gaussian–Laplacian pyramid decomposition	2019
	DRN [93]	ACM	multi-stage residual network with two residual blocks	2019
	NCANet [88]	Equation (A10)	rain streaks as residuals sum; recurrent	2022
	PRRNet [91]	ACM	stereo; semantic segmentation; multi-view fusion	2021
	GTA-Net [105]	ACM	multi-stream coarse; single-stream fine	2021

## 4. A Review of the Desnowing Literature

Sometimes, FRs need to detect victims in snowy environments, as for example mountain rescuers that search for victims who are lost in mountain areas. Snow is a common weather condition that can reduce the visibility of objects and scenes and obstruct the FRs’ missions. Unlike other atmospheric particles, snow particles have more complex characteristics, opaqueness, different shapes and sizes, uneven densities and irregular falling trajectories, which make the snow removal problem, also known as desnowing, more challenging [29].

### 4.1. Related Work on the Desnowing Problem

In this section, we review the published literature on the desnowing problem; that is, image restoration methods which are designed to remove snow particles from images. Table 5 lists the related work that we survey. Unlike the deraining and dehazing problems which have attracted significant attention by scientists, the desnowing problem has only attracted few research works. Importantly, however, researchers working on this problem also contributed datasets for use by the research community. Here we list the reviews on the related literature that we have collected on DL-based methods for single image desnowing.

#### 4.1.1. CNN-Based Desnowing Methods

Chen et al. [30] employ the Dual Tree Complex Wavelet Transform (DTCWT) to decompose an input image into a series of high-frequency (HF) and corresponding low-frequency (LF) components and propose the hierarchical DTCWT desnowing network (HDCW-Net). Each component is then recursively analyzed in terms of its HF and LF content, until *i* recurrence levels are reached. Then an HF reconstruction neural network is employed to reconstruct the HF image content, hence alleviating the effect of snow streaks and the snow veiling effect.

#### 4.1.2. Generative Models for Desnowing

**Figure 5:**
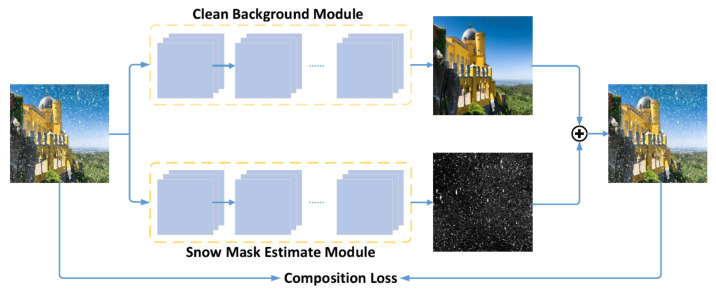
Schematic of the method by Li et al. [106]. A GAN-based architecture learns to extract the clean background image component and the rain streak layer component from an input rainy image by means of the composition loss function.

Li et al. [106] uses a compositional Generative Adversarial Network (compositional GAN) architecture to separate the clean background image regions and the regions contaminated by snow. This becomes possible by mainly employing the compositional loss, and some other minor loss functions that control the ability of the network to generate the correct output, to guide training of the cGAN model. As a model employing a GAN-style architecture, it contains a discriminator and a generator network. Figure 5 shows the schematic of the method.

**Figure 6:**
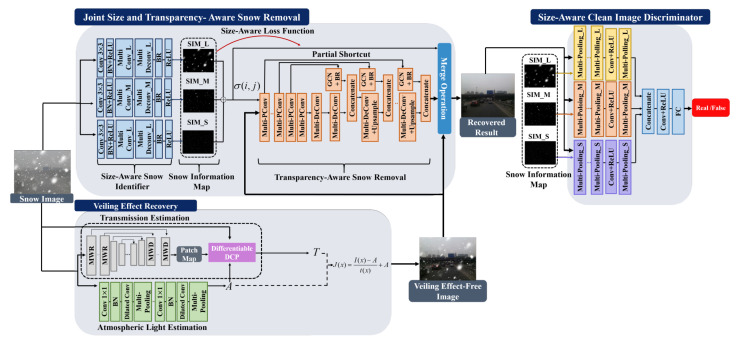
Schematic of the JSTASR method by Chen et al. [32]. The model comprises three modules. First, it contains a joint size and transparency-aware snow removal module. Secondly, it employs a veiling effect recovery module, and finally it comprises a size-aware clean image discriminator.

Chen et al. [32] proposes the JSTASR model (see Figure 6). This model can simultaneously handle transparent and non-transparent snow particles by applying the modified partial convolution. The model is transparency-aware; hence, it can handle snow with different transparencies. JSTASR can classify snow particles by considering their size and scale.

**Figure 7:**
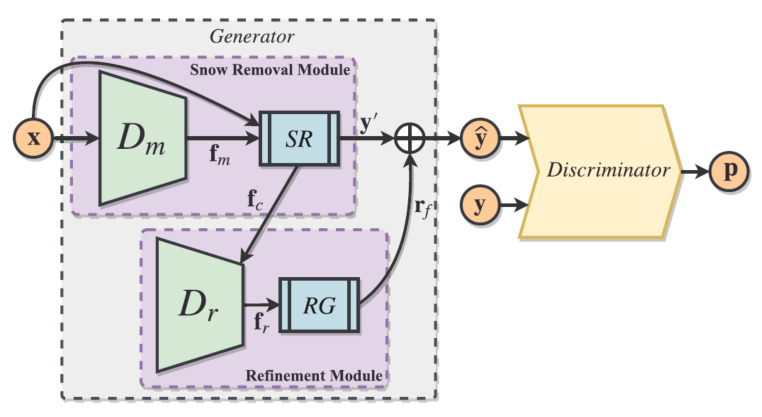
Schematic of the DesnowGAN method by Jaw et al. [107]. A GAN-based snow removal module, a refinement module and a discriminator module guide single image desnowing.

Jaw et al. [107] proposed a novel Deep Neural Network (DNN) architecture with a top-down pathway and lateral cross-resolution connections; it is depicted in Figure 7. The model (called DesnowGAN) exploits high-level semantic features and low-level spatial features for improved efficiency and running time. A novel loss function helps to jointly learn sharpness, structure and realism.

#### 4.1.3. Multi-Scale Based Desnowing Methods

**Figure 8:**
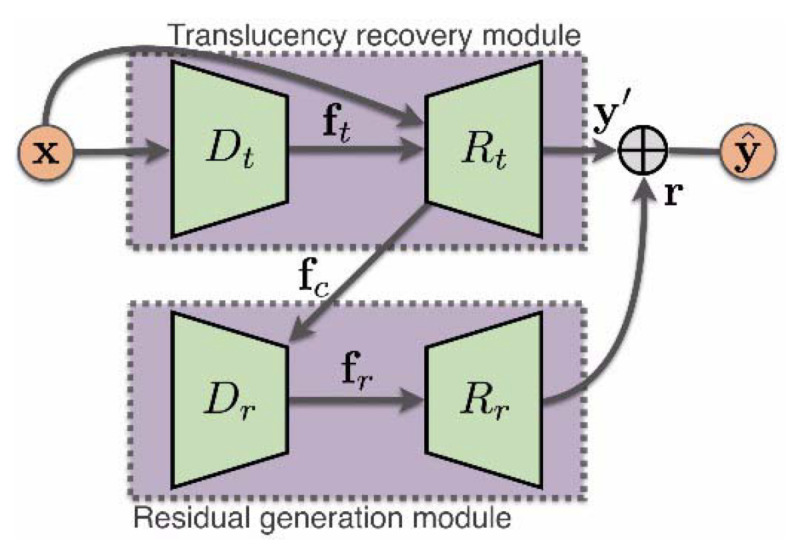
Schematic of the DesnowNet method by Liu et al. [29].

Liu et al. [29] proposes the DesnowNet model which deals with the removal of translucent and opaque snow particles instead of paying attention to the transparency of snow particles (see Figure 8). The model corrects the image content by accurately estimating and restoring details in the image that are lost due to opaque snow particles. It models snow by means of a snow mask, which considers only the translucency of snow at each coordinate, and also by means of a chromatic aberration map that captures fine color distortions. The model interprets snow through context-aware features and loss functions. DesnowNet also implements multi-scale receptive fields.

Li et al. [31] introduces a physics-based snow model and proposes a novel snow removal method based on the snow model and deep neural networks. The model decomposes a snowy image non-linearly as a combination of a snow-free image and dynamic snowflakes. The authors designed the Multi-scale Stacked Densely Connected Convolutional Network (MS-SDN) to simultaneously detect and remove snowflakes in an image. The MS-SDN is composed of a multi-scale convolutional subnetwork for extracting feature maps and two stacked modified DenseNets for snowflake detection and removal.

Zhang et al. [108] proposed the Deep Dense Multi-Scale Network (DDMSNet) for single image desnowing. The model can learn multi-scale representation off from pixel-level and feature-level input. Dense connections are applied to connect together multi-scale feature-computing subnetworks. The network exploits semantic and geometric information as prior knowledge. Semantic and geometric features are obtained in different stages to help recover snow and recover clean images.

**Table 5:** Listing of references on the desnowing problem.

Category	Method	Short Description	Year
CNN- based	HDCW-Net [30]	DTCWT analysis; recursively computes HF component; neural network reconstructs the last HF component	2021
Generative models	cGAN [106]	separates the background from snowy regions; uses compositional loss	2019
	JSTASR [32]	handles transparent/non-transparent snow particles; modified partial convolution; transparency aware; considers size and scale of snow particles	2020
	DesnowGAN [107]	DNN with top-down pathway and lateral cross-resolution connections; high-level and low-level spatial features; split-transform-merge topology reduces model size and computational cost; atrous spatial pyramid pooling for multi-scale and global receptive field	2020
Multi-scale based	DesnowNet [29]	accurately corrects image content by estimating and restoring details in the image that are lost due to opaque snow particles	2018
	MS-SDN [31]	multi-scale convolutional subnetwork extracts feature maps; stacked modified DenseNets for snowflakes detection and removal	2019
	DDMSNet [108]	multi-scale representation from pixel-level and feature-level input; multi-scale subnetwork are desnely connected; semantic and geometric priors; multistage analysis	2021

## 5. A Review of the Dehazing Literature

Haze can be defined as the atmospheric phenomenon where dust, smoke or other particles absorb and scatter the light, resulting in reduction of transparency of the air and consequently reduction of visibility [109]. FRs often need to take action in scenes of crises where their visual perception is affected by haze (fire, explosion, rescuing in the mountains, etc.), causing serious problems in the detection and navigation to possible victims.

Images captured in hazy environments suffer from loss of contrast, color fidelity and edge information which can further cause reduction of the performance of CV algorithms used for tasks like object detection, image segmentation, etc. Haze removal, which is referred to as dehazing, is considered an important process. Since most CV models assume clear weather conditions [110]. However, it can also be considered as a challenging problem, since the haze is dependent on the unknown depth information which varies at different positions [111].

### 5.1. A Taxonomy of the DL-Based Single Image Dehazing Methods

In order to solve the dehazing problem numerous methods have been proposed in the bibliography. These methods can be divided in two main categories: The hand-crafted priors-based methods and the data-driven methods. The hand-crafted priors-based methods use handcraft priors from empirical observation in order to remove the haze. They include priors such as dark channel (DCP) [111], contrast maximization [112], color attenuation [113] and non-local prior [114]. Unlike the hand-crafted priors-based methods, the data-driven methods use large-scale datasets to learn automatically the image prior. The main focus of this paper will be in data-driven methods. A synopsis of the methods is presented in Table 6.

#### 5.1.1. CNN-Based Dehazing Methods

Early data-driven methods include ML algorithms like random forest regressor [115], linear models [116] and the back propagation neural network [117]. In 2016, Cai et al. [118] adopted a trainable three-layer CNN to directly estimate the transmission map (for more information, see Section 6.3.5) from a hazy image and also developed the activation function called Bilateral Rectified Linear Unit (BReLU) to extract haze-relevant features for transmission recovery. Their pioneering model is known as Dehazenet and achieved a dramatically high efficiency compared to the state of the art of its times.

Li et al. [119] used CNNs in order to generate the clear image but their approach was different in the way that they wanted to avoid the estimation of the transmission map and the atmospheric light intensity. They introduced a new K(x) parameter that integrates these two parmeters in one as shown in Equation (2). Their model is known in the bibliography as All-in-One Dehazing Network (AOD-Net).

(1) J(x)=K(x)I(x)−K(x)+b

where *b* is the constant bias with the default value 1 and

(2) $$\begin{array}{c} K\left(x\right)=\frac{\frac{1}{t(x)}\left(I\left(x\right)-A\right)+\left(A-b\right)}{I(x)-1} \end{array}$$

Ullah et al. [120] exploited the transformed Atmospheric Scattering Model (ASM) proposed in [119]. They introduced a lightweight CNN architecture for estimating the K(x) parameter, that consists of eight convolution layers and three concatenation layers and an additional Color Visibility Restoration (CVR) module that helps recovering color information and contrast of the image by averaging color intensity and equalizing per channel histogram.

Qin et al. [121] considered two important facts in their model: (1) the haze distribution may be uneven on different pixels of the image; (2) the different channel features have totally different weighted information. Their proposed end-to-end Feature Fusion Attention Network (FFA-Net) uses both pixel attention and channel attention modules and combines them to design a feature attention module. The combined model is added to stacked residual blocks. In this way, they managed to focus on processing pixels with thick haze and more important channel information.

Inspired by the FFA-Net, Wu et al. [122] proposed an AE-like dehazing network with feature attention block as the basic block, the AECR-Net. They managed to make their network much more compact than the FFA-Net and also introduced a novel contrastive regularization in order to force the network to learn that the output dehazed image should be closer to the clear image and farther from the hazed one in the representation space.

#### 5.1.2. Multi-Scale Based Dehazing Methods

Inspired by the feature fusion structure of [121], Hu [123] proposed a multi-scale grid network (MSFFA-Net) in order to avoid the bottleneck issue happened in the conventional multi-scale approach.

Liu et al. [124] had a CNN-based approach to the problem, without taking the ASM into consideration. They introduced the GridDehazeNet (GDNet), a network consisting of three modules: the pre-process module which is a convolutional layer and a residual dense block and generates multiple different versions of the input image, the backbone module which performs attention-based multi-scale estimation based on the generated inputs and finally the post-processing module, which is a residual dense block following by a convolutional layer and achieves reduction of the artifacts.

Ren et al. [125] employed a Multi-Scale CNN (MSCNN), where firstly a coarse-scale network was used to learn the mapping between hazy inputs and their transmissions, and then a fine-scale network performs refined transmission estimation. The two networks are alternately merged and upsampled to maintain the original resolution. Extending their work, in 2020, Ren et al. [126] introduced an improved version of the MSCNN with fewer pooling and up-sampling layers in the coarse-scale network and a novel holistic edge guided network (MSCNN-HE) that ensures that the objects with the same depth have the same transmission values. Similarly, in Wang et al. [127] also followed the multi-scale coarse-to-fine idea and proposed an Ensemble Multi-scale Residual Attention Network (EMRA-Net). Their coarse network is a Three-scale Residual Attention CNN (TRA-CNN), where the different scales of the input image are produced using the 2-D Discrete Wavelet Transform (DWT). Their refined network is an Ensemble Attention CNN (EA-CNN) that fuses the three outputs of the coarse network into a final haze-free image.

Dong et al. [128] introduced the Multi-Scale Boosted Dehazing Network with Dense Feature Fusion (MSBDN). Their network is based on an encoder–decoder architecture with a dense feature fusion module and incorporates the Strengthen-Operate-Subtract (SOS) boosting strategy in the decoder to boost features of different levels. It also incorporates an error feedback principle in order to preserve the spatial information and exploit the features from non-adjacent levels.

Zhang et al. [129] adopted the AOD-Net’s transformed ASM proposed in [119] and tried to estimate K(x) fast and accurately in order to produce the final haze-free image according to Equation (2). Their proposed Fast and Accurate Multi-scale End-to-end Dehazing Network (FAMED-Net) has adopted the Gaussian/Laplacian pyramid architectures followed by a fusion module. Specifically, the input images are down-sampled to two different scales. The original and the two down-sampled images are fed to three encoders (one encoder for each scale) that do not share features. The outputs of the encoders are interpolated to the original scale and fed to a fusion module that estimates the K(x).

Zhao et al. [130] exploited long-range dependencies in order to improve the haze-free results. They introduced a Pyramid Global Context (PGC) block plugged into a U-Net, which is further improved by a dilated residual bottleneck (DRB) block. Their proposed network achieved to extract long-range dependencies among points and patches computationally more efficiently than by just stacking many convolutional layers.

In a recent work, Sheng et al. [131] considered the influence of the haze in the luminance of an image in the CIELAB colorspace. They proposed the multi-scale residual attention network (MSRA-Net), a network that consists of two subnetworks: one for the luminance and one for the chrominance. The network also embodies the Multi-Scale Residual Attention block (MSRA-block) and the Feature Aggregation Building block (FAB-block) and manages to improve the quality of the colors in the haze-free results.

Fan et al. [132] incorporated depth information in their multi-scale network (MSDFN). Specifically, they relied on the U-Net architecture. The depth image is first encoded and then decoded so as to produce the final haze-free image, while both in the encoding and decoding procedures the multi-level features of the hazy image are concatenated.

**Figure 9:**
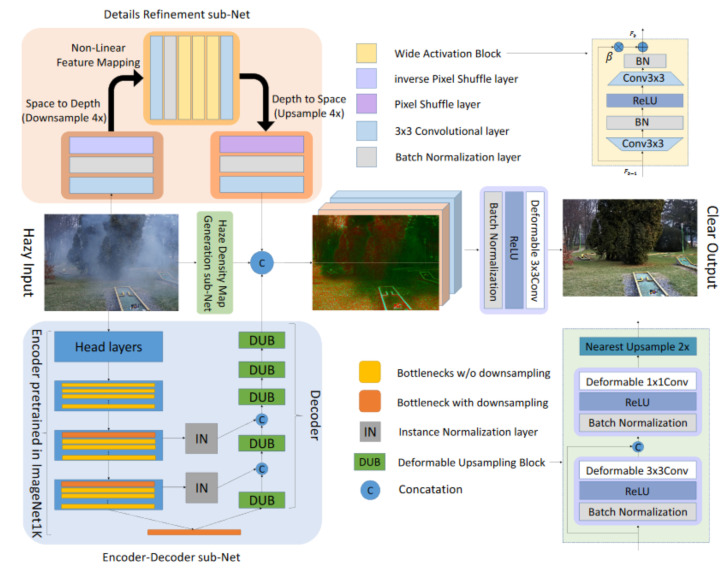
The architecture of Trident Dehazing Network [134]. ⊕ refers to tensor addition and ⊗ to tensor multiplication.

Das and Dutta [133] experimented with a deep multi-patch and a deep multi-scale network and tried to build a network which could remove the haze fast and efficiently even if it is non-homogeneous. They deduced that their multi-patch hierarchical network (DMPHN) is faster and better than their multi-scale hierarchical network, because it aggregates local features generated from a finer to a coarser grid. The Trident Dehazing Network (TDN) proposed by Liu et al. [134], depicted in Figure 9, also tries to remove efficiently the dense and non-homogeneous haze by following a multi-scale approach. It consists of three subnetworks: an encoder–decoder network that builds the first version of the haze-free image, a detail refinement network that focuses on the HF details and a haze density map generation network that detects which regions of the image have thick and which thin haze. Non-homogeneous and dense haze was also considered by Jo et al. [135]. They ignored the ASM and created a multi-scale architecture that employs the selective residual blocks (SRB) as its main functional module.

#### 5.1.3. Generative Models for Dehazing

Zhang et al. [136] proposed a dehazing GAN that takes the ASM into consideration, known in the bibliography as DCPDN. The generator consists of two subnetworks: an edge-preserving pyramid densely connected encoder–decoder network to estimate the transmission map and an 8-block U-net to estimate the atmospheric light. By using Equation (13), they obtained the dehazed image. They also introduced a new edge-preserving loss function to optimize the network that estimates the transmission map and a joint-discriminator based GAN framework to learn the dependencies between the dehazed image and the corresponding estimated transmission map. In [137,138], GANs are also used for image dehazing and depend on the ASM; however, the model in [138] is based on unpaired supervision.

Ren et al. [139] completely ignored the ASM. Their proposed Gated Fusion Network (GFN) is based on the fusion based encoder–decoder architecture, employed an original hazy image and three derived images as inputs (white balance, contrast enhancement, and gamma correction methods) and learned to predict confidence maps. After that, the confidence maps were fused to give the dehazed image. Their model was trained with MSE and adversarial loss. Qu et al. [140] also tried to avoid ASM and exploited an Enhanced Pix2pix Dehazing Network (EPDN). They utilised the idea of a multi-resolution generator, a multi-scale discriminator and an enhancer.

Li et al. [141] proposed an end-to-end trainable conditional GAN (cGAN) with encoder–decoder architecture that takes pairs of haze and haze-free images and restores directly the dehazed images without the need of ASM. Kan et al. [142] also exploited the paired cGAN framework and the multi-loss function optimization idea. They proposed a U-connection Residual Network (UR-Net) as a generator and the spatial pyramid pooling (SPP) structure for the discriminator. The multi-loss function is composed of a combination of adversary loss, $L_{1}$ loss, the structural similarity index (SSIM) loss and a new peak-signal-to-noise ratio (PSNR) loss. Another innovation of this work is that it offers a flexibility in the size of the input image by embedding the SPP structure.

**Figure 10:**
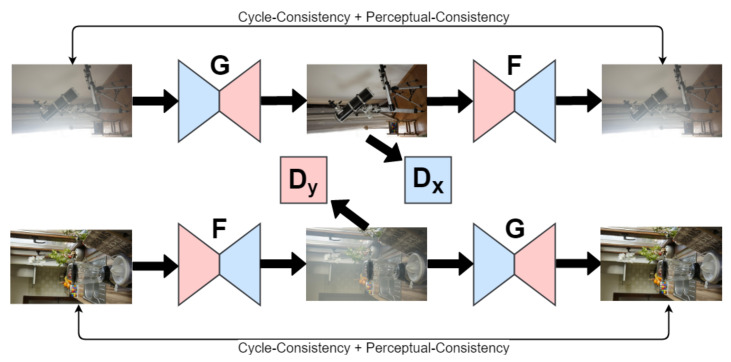
The architecture of the Cycle-Dehaze Model [143]. G and F are the generators, and D_x and D_y the discriminators.

Engin et al. [143] exploited the CycleGan architecture and enhanced it to recover the haze-free image from a hazed one. The model is depicted in Figure 10. The advantages of their so-called Cycle-Dehaze network are that it requires neither the estimation of the transmission map nor pairs of hazed and their corresponding clear images. The differences between CycleGan and Cycle-Dehaze are that Cycle-Dehaze uses an additional loss, the cyclic perceptual-consistency loss and the Laplacian pyramid as a post-processing step to upscale the dehazed images. Cycle-Dehaze achieves good dehazing results, however sometimes there exist some distortions.

Other works inspired by CycleGan architecture are [144,145,146,147]. In [144] Dudhane and Murala followed a similar training strategy as CycleGan, but their Cycle-consistent generative adversarial network (CDNet) differed in the architecture of the generator, where they introduced an encoder–decoder architecture with skip connections that estimates the transmission map. Liu et al. [145] proposed the Cycle-Defog2Refog network that consists of two transformation paths (hazy to haze-free, haze-free to hazy) and a two-stage mapping strategy in each transformation path so as to improve the dehazed results. In order to produce the synthetic hazy images they estimated the transmission map using a CNN approach. Jin et al. [146] was inspired by the CycleGAN architecture and added a Conditional Disentangle Network (UCDN), in order to manage different concentrations of haze. Mo et al. [147] wanted their network to have the ability to handle uneven and dense haze concentration and introduced the Dark Channel Attention optimized CycleGAN (DCA-CycleGAN). The DCA-CycleGAN consists of two subnetworks that compose the generator, two global discriminators and two local discriminators. The one subnetwork of the generator (Dark Channel Attention subnetwork) outputs attention maps that assist the other subnetwork (AE) to be fine-tuned and produce the dehazed image. Additionally, the local discriminators proposed in this work help the network to manage different concentration of haze in the image.

Park et al. [148] tried to fuse heterogeneous GANs and specifically the CycleGAN with the cGAN architecture. The concept of their work is to combine the great color balance that CycleGAN offer with the preservation of spatial details provided by cGANs. The CycleGAN is trained on unpaired, real, outdoor hazy and haze-free images, and learns to produce a haze-free result. The cGANs are trained on paired, synthesized indoor images. In their proposed method they exploited two different cGANs: the one learns to estimate the transmission map while the other learns to estimate the atmospheric light. Using Equation (13) they produce a second version of the haze-free result. After that, the two estimations of the haze-free results are fused in a CNN, which consists of feature extraction layers, a merge layer, and reconstruction layers.

Dong et al. [109] adopted the FD-GAN method, which is a GAN with a densely connected encoder–decoder as the generator and a fusion-discriminator that integrates HF and LF information as additional priors and constraints. Unlike Dong et al. [109], who use a fusion-discriminator, Fu et al. [149] suggested fusing the HF domain features into the generator. Their generative adversarial network (DW-GAN) uses a two-branch-designed network as a generator, where the one branch (DWT Branch) embeds the 2D DWT, so as to acquire clear texture details and the other (Knowledge Adaptation Branch) uses the pre-trained Res2Net as an encoder in order to avoid over-fitting.

In Wang et al. [150] introduced the TMS-GAN. Their architecture is based on two subnetworks: the Haze-generation GAN (HgGAN) and the Haze-removal GAN (HrGAN). The HgGAN takes a synthetic hazy image as input and learns to output a synthetic but realistic hazy image. The HrGAN learns to remove the haze both from synthetic and synthetic but realistic hazy images. Its generator is trained by the paired synthetic and synthetic but realistic data and achieves to produce a color-branch and detail-branch output. The element-wise addition of these two outputs and the clear image produced by the generator of HgGAN feed the discriminator of HrGAN so as to learn to produce the final haze-free images. Additionally, they proposed a plug-and-play Multi-attention Progressive Fusion Module (MAPFM) for both HgGAN and HrGAN.

#### 5.1.4. Deep Reinforcement Learning for Dehazing

Zhang and Dong [151] wanted to combine the simplicity of prior based methods with the generalization ability of DL and followed an innovative methodology in the image dehazing problem. They proposed a deep reinforcement learning (RL) based method (dehaze-RL), where a deep Q-learning network iteratively chooses actions in order to produce the final haze-free image. The actions set of the agent includes 11 actions that are existing dehazing algorithms and PSNR and SSIM metrics are used in the reward function as the measurement. The main advantage of this method is that the dehazed result is much more interpretable since in every state the corresponding result can be acquired.

Guo and Monga [152] considered the depth information of the image and proposed a Depth-aware Dehazing using RL system (DDRL). DDRL works by following the assumption that haze is lighter closer to the camera and denser farther from it, and consists of two networks: a policy network that generates the depth slice and a dehazing network that estimates the transmission map of each slice.

#### 5.1.5. Knowledge Distillation/Transferring for Dehazing

Hong et al. [153] presented a model that uses knowledge distillation to apply dehazing (KDDN), where the teacher is an auto-encoder that receives clean images, learns to reconstruct them and transfers the knowledge from the intermediate representations to the student network. The lightweight student network receives the hazy images and by mimicking features from the teacher learns to output the haze-free image. The student network’s supervision is based solely on dark channels loss and total variation loss.

Shao et al. [154] considered the domain shift problem. Usually, the models proposed for dehazing do not perform well in dehazing real hazy images, because they are trained on synthetic ones. Hence, they introduced a domain adaptation framework, which includes an image translation network and two dehazing networks (one for the synthetic domain and one for the real). In this way, they managed to reduce the domain discrepancy and achieve a good performance in the dehazing task in both domains. Domain gap was a problem that Chen et al. [155] also tried to address. They suggested a Principled Synthetic-to-real Dehazing (PSD) framework, which consists of a pre-trained network, trained on synthetic data as a backbone and they fine-tuned it in an unsupervised manner using real hazy images and physical priors that guide the fine-tuning process.

Yu et al. [156] recently introduced a two-branch neural network that is able to remove non-homogeneous haze and perform well even when trained on a small-scale dataset. The first subnetwork is a transfer learning subnetwork that extracts global representations from a pre-trained model, while the second one is trained from scratch on current training data and complements the first one. The final output is produced by ensembling the two subnetworks.

#### 5.1.6. Unsupervised/Semi-Supervised Learning for Dehazing

Overall, the CycleGAN-based networks described in Section 5.1.3 are trained in an unpaired manner. Yang et al. [138] was the first to adopt this unpaired training by introducing the conditional GAN architecture. Motivated by this idea, Golts et al. [157] evolved it and proposed a fully convolutional network with six dilated residual blocks. Their network aims to approximate the Dark Channel Prior (DCP) energy function and is trained using only hazy images. Additionally, an early stopping approach was followed, where a set of 500 paired images was used as a validation set and the training stopped in the epoch in which the validation set had the best performance in terms of PSNR and SSIM metrics.

Li et al. [158] presented a semi-supervised learning algorithm to solve the problem of single image dehazing. Their CNN-based network consists of two branches sharing the same weights and having the same encoder–decoder architecture: a supervised and an unsupervised one. The supervised branch is trained using synthetic, paired data and supervised losses while the unsupervised branch is trained using only real hazy images and unsupervised losses based on image priors.

RefineDNet proposed by Zhao et al. [159] combines the advantages of both prior-based and learning-based methods and requires unpaired hazy and haze-free images. Their two-stage network adopts the DCP in the first stage for visibility restoration and adversarial learning in the second stage for realness improvement. In order to improve the quality of the dehazed image even more, a perceptual fusion strategy is followed.

The You Only Look Yourself (YOLY) network proposed in [160] employs three joint disentanglement subnetworks: the J-Net, the T-Net and the A-Net that are designed to predict the clear image, the transmission map and the atmospheric light, respectively, in a self-supervised manner, given the hazy image as input.

**Table 6:** Listing of the surveyed research papers and articles on the dehazing problem.

Category	Method	Short Description	Year
CNN- based	Dehazenet [118]	3-layer CNN, BReLU activation function	2016
	AOD-Net [119]	lightweight, transformed ASM	2017
	Light-DehazeNet [120]	lightweight, transformed ASM, CVR module	2021
	FFA-Net [121]	attention-based feature fusion structure	2020
	AECR-Net [122]	AE-like, contrastive learning, feature fusion	2021
Multi-scale based	MSFFA-Net [123]	multi-scale grid network, feature fusion	2021
	GDNet [124]	3 sub-processes, multi-scale grid network	2019
	MSCNN [125]	2 nets: coarse- and fine-scale	2016
	MSCNN-HE [126]	3 nets: coarse-, fine-scale and holistic edge guided	2020
	EMRA-Net [127]	2 nets: TRA-CNN and EA-CNN	2021
	MSBDN [128]	dense feature fusion module, boosted decoder	2020
	FAMED-Net [129]	3 encoders at different scales, fusion module	2019
	PGC [130]	PGC and DRB blocks	2020
	MSRA-Net [131]	CIELAB, 2 subnets (luminance, chrominance)	2022
	MSDFN [132]	depth-aware dehazing	2021
	DMPHN [133]	non-homogeneous haze, multi-patch architecture	2020
	TDN [134]	3 subnets: coarse-, fine-scale and haze density	2020
	Jo et al. [135]	selective residual blocks	2021
Generative models	DCPDN [136]	generator with 2 subnets, edge-preserving loss function	2018
	DehazeGAN [137]	ASM-based GAN	2018
	DDN [138]	ASM-based, unpaired supervision	2018
	GFN [139]	fusion based, employs a hazy image and 3 derived inputs	2018
	EPDN [140]	multi-resolution generator, multi-scale discriminator, enhancer	2019
	cGAN [141]	cGAN with encoder–decoder architecture	2018
	Kan et al. [142]	cGAN, UR-Net as a generator, flexibility in image size	2022
	Cycle-Dehaze [143]	CycleGan based, unpaired supervision	2018
	CDNet [144]	CycleGan based, encoder–decoder architecture for the generator	2019
	Cycle-Defog2Refog [145]	2 transformation paths with 2-stage mapping strategy in each	2020
	UCDN [146]	CycleGan based with a conditional disentangle network	2020
	DCA-CycleGAN [147]	generator with 2 subnets, 4 discriminators	2022
	Park et al. [148]	fusion of cGAN and CycleGAN	2020
	FD-GAN [109]	integration of HF and LF information in the discriminator	2020
	DW-GAN [149]	generator with a DWT and a Knowledge Adaptation Branch	2021
	TMS-GAN [150]	2 subnets: a haze-generation and a haze-removal GAN	2021
RL-based	Dehaze-RL [151]	actions: 11 dehazing algorithms, reward function: PSNR and SSIM	2020
	DDRL [152]	depth-aware dehazing	2020
Knowledge distillation/ transferring	KDDN [153]	teacher-student (dehazing) net	2020
	Shao et al. [154]	domain adaptation using a bidirectional translation net	2020
	PSD [155]	domain adaptation by unsupervised fine-tuning (real domain) a pre-trained model (synthetic domain)	2021
	Yu et al. [156]	2-branch net: transfer learning and current data fitting subnets	2021
Unsupervised/ Semi- supervised	Golts et al. [157]	unsupervised, DCP loss	2019
	Li et al. [158]	2-branch: supervised and unsupervised subnets	2019
	RefineDNet [159]	2-stage network: DCP and adversarial learning stages	2021
	YOLY [160]	self-supervised, 3 joint disentanglement subnetworks	2021

### 5.2. Multi-Image Dehazing

Other modalities in the DL-based dehazing literature (apart from the single image) include video dehazing and binocular image dehazing.

In the binocular, DL-based image dehazing few methods have been proposed recently. Song et al. [161] proposed an encoder–decoder architecture that jointly learns to improve the quality of the disparity maps and the dehazing results. Pang et al. [162] tried to avoid the time consuming estimation of the disparity map and proposed the BidNet, which receives binocular image pairs and try to dehaze them by exploring the correlation between the images using the Stereo Transformation Module. The most recent Stereo Refinement Dehazing Network (SRDNet) [163] follows a coarse-to-fine approach and tries to remove the haze of the binocular image pairs by avoiding exploiting the ASM. Their proposed framework consists of two networks: a weight-sharing coarse dehazing network and a guided separated refinement network, where the latter fuses the information from cross views avoiding in this way the estimation of the disparity or correlation matrix.

Although, in the literature, the single image dehazing algorithms are often used for video dehazing, video dehazing can also be considered as a separate task, since the temporal coherence of video frames offers a great amount of useful information that cannot be found in single images. Li et al. [164] employed a CNN-based architecture for video dehazing. Their End-to-End Video Dehazing Network (EVD-Net) is based on the AOD-Net [119] architecture and follows three different types of temporal fusion structures in order to consider the available information of the temporal coherence of video frames. Ren et al. [165] proposed an encoder–decoder architecture that collects information across frames in order to estimate the transmission map. Their network tries to restore the frames with the same scenes in a way that the transmissions are smooth enough using two different fusion strategies.

## 6. Results

### 6.1. Quantitative Metrics

In order to evaluate the quality of the output image produced by a deraining, a desnowing or a dehazing model, there are two widely used metrics. These metrics compare the differences between an output and a reference image and for that reason they are also called full-reference metrics. In this section, we mention the two most common quantitative metrics that are commonplace in every possible study on deraining, desnowing and dehazing.

#### 6.1.1. Peak Signal-to-Noise Ratio

The peak-signal-to-noise ratio (PSNR) measures the discrepancy among two images *I* (a reference image) and *K* (a ground-truth image). The discrepancy is measured numerically as the log (base 10) of the ratio of the square of the maximum intensity value in the image *I* to the mean squared error (MSE) of the corresponding pixel intensity values in image *I* and image *K*. Formally, the PSNR measure is given by the formula

(3) $$PSNR=10\log_{10}\left(MAX_{I}^{2}/MSE\right)$$

where $MAX_{I}$ is the maximum intensity value in the set of pixels in image *I* and MSE is the value of the mean squared error of the pixel intensities among the reference image *I* and the ground-truth image *K*. The MSE value is given by the formula

(4) $$MSE=\frac{1}{mn}\sum_{i=0}^{m-1}\sum_{j=0}^{n-1}{\left[I\left(i,j\right)-K\left(i,j\right)\right]}^{2}$$

#### 6.1.2. Structural Similarity

The structural similarity index (SSIM) [166] is a metric that measures the perceptual difference between two images *x* and *y* and is given by

(5) $$SSIM\left(x,y\right)=\frac{\left(2\mu_{x}\mu_{y}+c_{1}\right)\left(2\sigma_{xy}+c_{2}\right)}{\left(\mu_{x}^{2}+\mu_{y}^{2}+c_{1}\right)\left(\sigma_{x}^{2}+\sigma_{y}^{2}+c_{2}\right)}$$

where $c_{1}={\left(k_{1}L\right)}^{2}$ and $c_{2}={\left(k_{2}L\right)}^{2}$ and *L* is the dynamic range of the pixel values defined as $L=2^{bitsperpixel}-1$ and $k_{1}=0.01$ and $k_{2}=0.03$. $\mu_{x}$ and $\mu_{y}$ are the expected values of the variables *x* and *y*, and $\sigma_{x}$ and $\sigma_{y}$ are the variances of *x* and *y*.

### 6.2. Real-Time Performance Classification

Another critical aspect that must be considered when employing a DL algorithm for enhancing the situational awareness of FRs is whether they are able to process data quickly. However, reports on different methods often regard different image resolution, which naturally has a significant impact on execution time. Hence, in the comparisons of this section, we have classified each method as real-, near-real- or non-real-time, taking into consideration both reported frames per second (FPS) and image resolution. The real-time category includes methods that report high FPS in at least medium resolutions, or medium FPS in higher resolutions. Near-real-time methods may report medium FPS in medium resolutions or lower, but still acceptable FPS, in high resolutions. With improving hardware and computational advances, near-real-time methods may achieve higher FPS and be promoted to real-time in the near future. Non-real-time methods report very low FPS. It must be stressed that this classification is still subjective and should not be interpreted as a strict classification scheme.

### 6.3. Comparison of Models

The deraining, desnowing and dehazing methods that could serve the purpose of augmenting the vision of FRs during rescue mission operations should have a low response time and produce a visually pleasing output. Therefore, in this section we discuss the results of the models proposed in the literature for deraining, desnowing and dehazing in terms of PSNR and SSIM values, as well as their processing time. The compared methods are evaluated based on benchmarking datasets. In Table 7, Table 8 and Table 9 we report the results for the deraining, desnowing and dehazing methods respectively. The highest PSNR and SSIM values are highlighted using bold text and the algorithms have been sorted in a top to bottom order, where the top is the method with the highest processing time needed.

Additionally, we have classified the methods in three categories (real-time, near-real-time, non-real-time) based on the maximum images per second that the models can process (for more information please see Section 6.2), considering also the size of the images. Due to the large number of algorithms found in the literature and the lack of availability of code for many of the methods, the reported results are only based on the literature and no experiments have been made by the authors of this paper. Lastly, some methods have been omitted due to lack of available information.

#### 6.3.1. Mathematical Background of Deraining

Mathematically, a rainy image *O* can be modeled as a linear superimposition of the clean background image *B*, and the rain streak layer *S* which contains the rain streaks that we would like to remove, as shown in Equation (6).

(6) O=B+S

By estimating *S* we can, in turn, recover the clean image *B* through the difference as shown in Equation (7).

(7) B=O−S

The problem of decomposing the rainy image to a clear image and a rain streak layer is an ill-posed problem and to resolve this many different approaches have been proposed. In Appendix A.1 we list the different imaging models that have been proposed for the image deraining task.

#### 6.3.2. Comparison of Deraining Models

In Table 7 we have collected the methods discussed in Section 3 and classified them in terms of quality metrics and processing time. We observe that the best quantitative performance is obtained by the JORDER [23] and ResGuideNet3 [55] methods. Their performance is justified by the reported PSNR and SSIM values. More specifically, the JORDER [23] method exhibits the highest PSNR value (namely, 32.95), while ResGuideNet3 [55] exhibits the highest SSIM value (estimated at 0.939). A drawback is that, while these two methods score high in terms of the PSNR or SSIM value, they both reach an average processing time and can be classified as near-real-time performance. Two of the methods have an exceptionally short processing time, namely the PCNet-fast [167] and the LPNET [86]. The MSPFN [80], IADN [71] and DDN [17] methods are observed to have an adequate performance but at the same time they perform in near-real-time settings. We can conclude that for an augmented vision application around rescue mission operations, a model selection strategy could be to select a method that has at least near-real-time processing time, and simultaneously a high value of the exhibited PSNR or SSIM. Using this principle we can select the PCNet-fast, PCNet or ResGuideNet3 methods. By assuming a high PSNR or SSIM value, we can potentially improve the processing time capabilities of a method so that it can be feasible to use it for the intended scenario. Such methods are, for instance, IADN, PReNet, MSPFN and RESCAN.

**Table 7:** Quantitative results of deraining methods along with their running time. ↑ means higher is better. In **bold** we indicate the best result for each dataset.

Dataset	Method	PSNR ↑	SSIM ↑	FPS ↑	Image Resolution	Classification
Test1200	RESCAN [87]	30.51	0.882	1.83	512×512	non-real-time
	MSPFN [80]	32.39	**0.916**	1.97	512×512	non-real-time
	PReNet [89]	31.36	0.911	6.13	512×512	near-real-time
	IADN [71]	**32.29**	**0.916**	7.57	512×512	near-real-time
	DDC [25]	28.65	0.854	8.00	512×512	near-real-time
	DerainNet [48]	23.38	0.835	13.51	512×512	near-real-time
	PCNet [167]	32.03	0.913	16.12	512×512	near-real-time
	UMRL [104]	21.15	0.770	20.00	512×512	real-time
	PCNet-fast [167]	31.45	0.906	35.71	512×512	real-time
	LPNET [86]	25.00	0.782	**37.03**	512×512	real-time
Rain100L	JORDER [23]	**32.95**	0.921	5.55	481×321	near-real-time
	DDN [17]	31.12	0.926	6.25	481×321	near-real-time
	ResGuideNet3 [55]	30.79	**0.939**	**16.66**	481×321	near-real-time

#### 6.3.3. Mathematical Background of Desnowing

Removing snow from a single image is an ill-posed problem. In the literature, it is often assumed that a snowy image is composed of a clear image and an independent snow mask and the problem is formulated using Equation (8)

(8) K(x)=J(x)(1−Z(x))+C(x)Z(x)

where $K\in {\left[0,1\right]}^{p\times q\times 3}$ is a colored snowy image of size p×q, $J\in {\left[0,1\right]}^{p\times q\times 3}$ is the corresponding snow-free image, $Z\in {\left[0,1\right]}^{p\times q\times 1}$ is the snow mask and *C* is the chromatic aberration map [29].

Recently, Chen et al. [32] reformulated this snow formation model, in order to additionally take the veiling effect into consideration. Their Joint Size and Transparency-Aware Snow Removal (JSTASR) model is inspired by the Atmospheric Scattering Model used for dehazing (for more information the reader can refer to Section 6.3.5) and is formulated using Equation (9)

(9) I(x)=K(x)T(x)+A(x)(1−T(x))

where *A* is the atmospheric light matrix, *J* is the scene radiance, *T* is the transmission map and *K* is the snowy image without the veiling effect defined as:

(10) K(x)=J(x)(1−Z(x)R(x))+C(x)Z(x)R(x)

where *R* is a binary mask which represents the snow location.

#### 6.3.4. Comparison of Desnowing Models

Table 8 lists the reported quality metrics and processing time throughput of the listed methods for desnowing discussed in Section 4. Again, in terms of execution time we use the same classification and the quantitative metrics are the PSNR and SSIM. Clearly, the DesnowGAN method by Jaw et al. [107] is the only method that exhibits real-time performance. This method proposes multiple model variations which are capable of generating a variable number of FPS. In particular, it can generate between 14 and 33 FPS. We keep the one with the highest number of FPS in the context of assisting FRs. The JSTASR [32], MS-SDN [31] and DesnowNet [29] methods belong to the class of non-real-time methods. It is also interesting to mention that the DesnowNet [29] and the MS-SDN [31] models score higher in PSNR and SSIM scores in contrast to the DesnowGAN [107] method. The latter method is very fast in terms of FPS, but it scores lower in PSNR and SSIM scores. In this case, the DesnowGAN [107] could be a good fit for enhancing the situational awareness of FRs due to its high speed, but the MS-SDN model [31] could also be an option if we consider improving its processing time.

**Table 8:** Quantitative results of desnowing methods along with their running time. ↑ means higher is better. In **bold** we indicate the best result among the evaluated methods.

Dataset	Method	PSNR ↑	SSIM ↑	FPS ↑	Image Resolution	Classification
Snow-100K (overall)	DesnowNet [29]	**30.11**	0.930	0.72	640×480	non-real-time
	MS-SDN [31]	29.25	**0.936**	2.38	640×480	non-real-time
	JSTASR [32]	28.61	0.864	2.77	640×480	non-real-time
	DesnowGAN [107]	28.18	0.912	**33.33**	640×480	real-time

#### 6.3.5. Mathematical Background of Dehazing

In CV and computer graphics, a hazy image is modeled mathematically using the Atmospheric Scattering Model (ASM) (Equation 11) proposed by McCartney [168].

(11) I(x)=J(x)t(x)+A(1−t(x))

where *x* represents the position of pixels, I(x) is the observed hazy image and J(x) the corresponding haze-free image to be recovered. The first term on the right hand side of Equation (11) J(x)t(x) is called direct attenuation, while the second term A(1−t(x)) airlight. There are two critical parameters in the ASM: the parameter *A* that denotes the global atmospheric light, and the parameter t(x) that is the transmission map, defined as in Equation (12).

(12) $$t\left(x\right)=e^{-\beta d(x)}$$

where β is the scattering coefficient of the atmosphere and d(x) is the distance between the object and the camera.

Hence, solving Equation (11) for *J(x)*, the model for the clean image can be written as

(13) $$J\left(x\right)=\frac{1}{t(x)}I\left(x\right)-A\frac{1}{t(x)}+A$$

In clear weather conditions β≈0; therefore, I≈J. In hazy images the *A* and *t* parameters need to be estimated.

#### 6.3.6. Comparison of Dehazing Models

In Table 9 we have collected the methods discussed in Section 5 and classified them in terms of quality metrics and processing time, based on the best performance found in the literature. The quality metrics used for evaluation are the PSNR and SSIM metrics and the dataset used for evaluation is the RESIDE—SOTS. As can be seen, the MSFFA-Net [123] has the best PSNR value (36.69), the method proposed by Yu et al. [156] has the best SSIM value (0.991), while the AOD-Net has the shortest processing time. There is a trend that the faster the model is the worse the metrics are, but there exist some exceptions. FAMED-Net [129] and FD-GAN [109] show a relatively good performance although they are real-time methods, while the MSCNN [125] is a near-real-time method but has poorer performance. Since for the specific task we are interested both in execution time and the quality of the output images the method proposed by Yu et al. [156] and the DW-GAN [149] could be a good fit, while finding a way to reduce their processing time is also a great idea for having a real-time algorithm with great quantitative metrics.

**Table 9:** Quantitative results of dehazing methods along with their running time. ↑ means higher is better. In **bold** we indicate the best result among the evaluated methods.

Dataset	Method	PSNR ↑	SSIM ↑	FPS ↑	Image Resolution	Classification
SOTS (RESIDE)	FFA-Net [121]	36.39	0.988	0.57	1600×1200	non-real-time
	Li et al. [158]	24.44	0.890	0.89	512×512	non-real-time
	MSCNN-HE [126]	21.56	0.860	1.20	427×370	non-real-time
	TDN [134]	34.59	0.975	1.58	1600×1200	near-real-time
	DW-GAN [149]	35.94	0.986	2.08	1600×1200	near-real-time
	Light-DehazeNet [120]	28.39	0.948	2.38	620×460	non-real-time
	PGC [130]	28.78	0.956	3.17	563×752	near-real-time
	MSFFA-Net [123]	**36.69**	0.990	3.23	620×460	near-real-time
	DehazeNet [118]	21.14	0.847	3.33	620×460	near-real-time
	EPDN [140]	25.06	0.923	3.41	563×752	near-real-time
	Golts et al. [157]	24.08	0.933	3.57	620×460	near-real-time
	GDNet [124]	32.16	0.983	3.60	620×460	near-real-time
	MSCNN [125]	17.57	0.810	3.85	620×460	near-real-time
	YOLY [160]	19.41	0.832	4.76	620×460	near-real-time
	Yu et al. [156]	36.61	**0.991**	11.24	1600×1200	real-time
	cGAN [141]	26.63	0.942	19.23	620×460	real-time
	GFN [139]	22.30	0.880	20.40	620×460	real-time
	DCPDN [136]	19.39	0.650	23.98	512×512	real-time
	FD-GAN [109]	23.15	0.920	65.00	1024×1024	real-time
	DMPHN [133]	16.94	0.617	68.96	1600×1200	real-time
	FAMED-Net [129]	25.00	0.917	86.20	620×460	real-time
	AOD-Net [119]	19.06	0.850	**232.56**	620×460	real-time

## 7. Discussion and Conclusions

DL algorithms for enhancing vision in adverse weather conditions have raised a lot of interest in the past few years. This state-of-the-art report presents the vast increase of research in this domain while focusing on the specific case of assisting FRs. We believe that such algorithms will have a profound impact on improving the situational awareness of FRs in cases where their vision is bound by natural phenomena. We first introduce commonly used datasets, next we present a taxonomy and detailed review of the three families of image restoration methods for adverse weather conditions based on the most recent research papers. Attention is also paid to unified image restoration methods that can simultaneously handle rain, snow and haze. These models can blindly understand which kind of weather-specific processing should be applied to an input image without requiring the user to specify in what weather condition the image was captured. Finally, we discuss the suitability of each of the existing algorithms for rescue missions applications. In order to decide the competence of each method, we present a comparison of them in terms of qualitative metrics and processing time. From our results, we can conclude that the real-time performance for such image restoration tasks is of paramount importance to integrating them into systems that can perform live in rescue missions.

Furthermore, there are very few real-world datasets and none of them are focused on the specific task of rescue missions, thus it will be necessary to create future datasets focused on rescue missions. We hope that this survey will introduce image restoration methods for FRs to a large research community, thereby facilitating the development of next-generation image restoration applications focusing on both execution time and performance. Last but not least, research directions such as unified models that operate under multiple adverse conditions can open new pathways for deploying robust vision applications that will be valuable both for researchers and experts.

## Data Availability

Not applicable.

## Abbreviations

The following abbreviations are used in this manuscript:

FR	First Responder
CV	Computer Vision
DL	Deep Learning
ML	Machine Learning
GPU	Graphics Processing Unit
CNN	Convolutional Neural Network
GAN	Generative Adversarial Network
AE	Autoencoder
HF	High Frequency
LF	Low Frequency
PDU	Progressive Dilated Unit
UBS	Unsupervised Background Segmentation
LSTM	Long Short-Term Memory
DNN	Deep Neural Network
DWT	Discrete Wavelet Transform
RL	Reinforcement Learning
PSNR	Peak-Signal-to-Noise Ratio
SSIM	Structural Similarity Index Measure
FPS	Frames Per Second

## Appendix A

### Appendix A.1. Underlying Equations for Image Deraining

This Appendix section supplements Section 6.3.1. Here we discuss several imaging models that have been proposed in methodologies on the deraining task. These imaging models, in general, make an attempt to separate the clean image data from the rain streak data in various ways. Due to the complex data structure that exists in real image data, the imaging model equations that have been proposed may not model the underlying data perfectly or perfectly separate the usable data from the undesired noise. Below, we list the synthetic imaging models inline along with the references which introduced them.

1. Li et al. [90] model the observed rainy image *O* as a background layer *B* and a sequence of *s* rain streak layers $S_{t}$. Each layer $S_{t}$ can model rain streaks of different characteristics (e.g., that of orientation and size). Their proposed model is known in the bibliography as Multiple Rain Streak Layers (MRSL) model and is shown in Equation (A1).
  (A1) $$O=B+\sum_{t=1}^{s}S_{t}$$
2. The Heavy Rain Model (HRM) proposed by Yang et al. [24] is a convex combination of two quantities: (1) the underlying image modelled by a set of rain streak layers $S_{t}$ (*s* layers are assumed) and the background image *B*; (2) the global atmospheric light matrix *A*. The *s* rain-streak layers $S_{t}$ are able to capture the rain streaks with different characteristics. *a* is the atmospheric transmission parameter. Equation (A2) shows the HRM model.
  (A2) $$O=\alpha ⊙\left(B+\sum_{t=1}^{s}S_{t}\right)+\left(1-\alpha \right)⊙A$$
3. The model proposed by Xue et al. [54] assumes an image B+R that is gamma corrected by an exponent γ. This image is linearly combined with the atmospheric light matrix *A*. This equation is similar to Equation (A2), except for the fact that the latter requires multiple rain streak layers $S_{t}$. Their proposed model is known in the bibliography as Heavy Rain Model with Low Light (HRMLL) model and is shown in Equation (A3).
  (A3) $$O=\alpha {\left(B+R\right)}^{\gamma }+\left(1-\alpha \right)A$$
4. Luo et al. [169] introduced the Screen Blend Model (SBM). Their model is inspired by the Equation (6), but a difference is that in the equation the last term B⊙S is subtracted from the quantity B+S. The entries in the matrix B⊙S weigh each pixel entry by the relative importance of the background pixel value of the associated entry and the corresponding value in matrix *S*. The entries of this matrix can be regarded as the linear correlation among the signals. Therefore, each pixel intensity associated with an entry of *O* is reduced by the product of the values from *B* and *S*. The model is shown in Equation (A4).
  (A4) O=B+S−B⊙S
5. The equation Rain Model With Occlusion (RMO) by Liu et al. [170] is similar to the HRM, except that they mutually differ in the last term (1−β)⊙R. In this term $\beta_{t(i,j)}$ is a cancellation term that signifies whether the pixel at location (i,j) belongs to the rain occluded region $\Omega_{s}$ (Liu et al. [170] defines $\Omega_{s}$ as “the region where the light transmittance of rain drop is low”) and matrix *R* is the rain reliance map.
  (A5) $$O=\beta ⊙\left(B+\sum_{t=1}^{s}S_{t}\right)+\left(1-\beta \right)⊙R$$
6. Yang et al. [103] introduced the Comprehensive Rain Model (CRM). This model is similar to HRM, however, here a matrix component *U* is added to the right factor of the first term of the equation in case 5. The CRM is shown in Equation (A6).
  (A6) $$O=\beta ⊙\left[\left(B+\sum_{t=1}^{s}S_{t}\right)+\left(1-\alpha \right)A+U\right]+\left(1-\beta \right)⊙R$$
7. The Depth-aware Rain Model (DRM) by Hu et al. [72] shares a similarity with the previous equations in that terms that appear in the previous equations also appear in this last equation but in a different order. The DRM is shown in Equation (A7)
  (A7) $$O=\beta ⊙\left(1-\sum_{t=1}^{s}S_{t}-\left(1-\alpha \right)A\right)+\sum_{t=1}^{s}S_{t}+\alpha A$$
8. Equation (A8) by Li et al. [87] combines linearly the clean background image *B*, the rain streak layers $R_{i}$ and the atmospheric light matrix *A*. This linear combination is especially a convex combination.
  (A8) $$O=\left(1-\sum_{i=0}^{n}a_{i}\right)B+a_{0}A+\sum_{i=1}^{n}\alpha_{i}R_{i}$$
9. Equation (A9) by Yang et al. [23] is similar to Equation (6). The difference in the former equation is that the entries of matrix *R* are linearly combined with cancellation entries in a matrix *S*. When an entry of *S* is zero, the respective entry in matrix *R* is cancelled. In turn, when an entry of *S* equals 1, then the respective intensity value in the entry of *R* is promoted. Hence, the term *O* in O=B+S⊙R is similar to the equation O=B+R, but a difference is that the entries in S⊙R where the respective entry in *S* is zero are cancelled. When each entry in *S* equals 1, the respective entry in *O* is modelled as O=B+R.
  (A9) O=B+S⊙R
10. Equation (A10) by Su et al. [88] involves the iterative estimation of the background image *B*. The equation asserts that the rain streak layer *R* is determined by the paired differences of estimated background images $B_{i}$ and $B_{i-1}$.
  (A10) $$\sum_{i=2}^{N}\left(B_{i}-B_{i-1}\right)=R$$
11. The Raindrop Equation (A11) proposed by Qian et al. [65] models the relationship between the colored image *I*, a binary mask matrix *M* with zero-or-one entries, the clean background image *B* and the matrix *R*. Matrix *R* represents the raindrop information, which is a combination of the background information and the light reflected by the environment through the raindrops.
  (A11) I=(1−M)⊙B+R
12. The Rain Accumulation and Accumulation Flow (RAAF) model (Equation (A12)) was introduced in the paper of Yang et al. [103]. The parameter $A_{t}$ is the global atmospheric light. $\beta_{t}$ is atmospheric transmission. $U_{t}$ is the rain accumulation flow layer, and $S_{t}$ is the rain streak layer.
  (A12) $$O_{t}=\beta_{t}B_{t}+\left(1-\beta_{t}\right)A_{t}+U_{t}+S_{t}$$

### Appendix A.2. Loss Functions Employed in the Deraining Problem

In this section, we review some notable loss functions which have been used by recent deraining methods.

The perceptual image similarity loss [171] measures the discrepancy of a derained image and the ground-truth of the image. To measure discrepancy, the loss function takes the difference between the feature activations of the derained image $x^{D}$ and the feature activations of the ground-truth image $x^{G}$. The components of the resulting feature activation vector are reweighted by a vector $\theta_{i}$.

The perceptual contrastive loss [61] is the weighted combination of *n* fraction terms referring to the *i*-th layer $V_{i}$ of an underlying CNN. Three quantities are considered in this loss function: the derained image $x^{D}$, the rainy image $x^{R}$ and the ground-truth image $x^{G}$. In the fraction terms, the nominator is the $\ell_{1}$ distance between the feature activations of the derained image and the ground-truth image. The denominator measures the distance between the feature activations of the *i*-th CNN network for the derained image $x^{D}$ and the rainy image $x^{R}$.

The segmentation loss used in [61] is also commonly known as the focal loss function [172]. The probability $p_{ij}$ models the likelihood that the pixel at position (i,j) belongs to a particular class, and *a* and γ are standard parameters. The function computes the average bit count corresponding to the probability value $p_{ij}$, each weighted by the factor ${-a(1-p_{ij})}^{\gamma }$. In this way, the supervision focuses on hard examples while reducing the loss contribution from easy examples.

The margin loss considers two extreme values $m^{+}$ and $m^{-}$ and measures their relation to the squared $\ell_{2}$ norm of a vector $v_{k}$. If we consider two points $m^{-}$ and $m^{+}$ on a straight line, then $\left|\right|v_{k}{\left|\right|}^{2}$ can be placed at three possible point locations. Based on this location, the max operators of $T_{k}$ and $1-T_{k}$ are either positive or are cancelled out, and the loss function varies among a minimum and a maximum value.

The color cycle-consistency loss used in [173] is a trivial modification of the cycle-consistency loss, originally introduced in [67]. In a Cycle-GAN, a network involving a generator and a discriminator function is considered. This network employs a cycle-consistency constraint which ensures that a datum that is projected via projection function *F* can be back-projected to the original vector space by another function *G*. The color cycle-consistency loss function applies the cycle consistency constraint on the three color channels of an RGB image matrix of reference.

The identity preserving loss used in [174] considers the absolute error of the coordinates of two vectors. The first vector is a vector *n* and the mapping $G_{N2R}\left(n\right)$, and the second vector is a vector *r* and its projection vector $G_{R2N}\left(r\right)$. The target in this loss function is to minimize the average value of the $\ell_{1}$ vector norm of the difference of the two pairs of vectors.

The total variation loss is the sum of the absolute differences for neighboring values of an input image. It contributes to generating more smooth images while removing the rough textures of the generated denoised image.

Adversarial loss [64] measures the distance between distribution of the generated images and the distribution of the ground truth images. In these settings, an adversarial network can have two loss functions, one for the generator who tries too fool the disciminator, and one for the discriminator who tries to classify a forged distribution from a real one.

The attention loss function used in [78] is one out of many loss functions designed for serving an attention mechanism in deep neural networks. This loss function considers the $\ell_{2}$ matrix norm of the difference of an attention-mask matrix referring to an *s*-th scale and a target attention matrix.

The Charbonnier loss function is a matrix function that involves the $\ell_{2}$ matrix norm of the input variable and a lag term ϵ that ensures the numerical stability of the loss function. Since the $\ell_{2}$ matrix norm of the input matrix variable is non-negative, the Charbonnier loss function adds a small parameter $\epsilon^{2}$ to the squared $\ell_{2}$ matrix norm that ensures that the final loss function value equals at least ϵ.

Structural similarity is used in the deraining problem as a surrogate loss function to optimize the weights of a deep neural network. Similarly, the peak-signal-to-noise ratio metric, which is also a no-reference metric for measuring the quality of an image, is also used as a surrogate loss function in some of the works on the deraining problem; for example, see [175]. Both functions are a natural choice of loss functions for a deep neural network capable of solving the single image deraining problem. Since in deep neural networks we seek to minimize the underlying loss function, we need to minimize the negative of either function. In fact, in practice we want to maximize terms involving the structural similarity and peak signal-to-noise, usually taken to be the mean structural similarity value and the mean peak signal-to-noise ratio.

### Appendix A.3. Loss Functions Used by Desnowing Methods

In this part of the Appendix, we list the loss functions that are introduced by the DL-based single image desnowing methods available in Section 4.1. Except simple cases of loss functions that are common in the general literature of DL, Table A1 lists several important loss functions which do not fall within the common loss function cases. Importantly, these non-trivial loss functions are themselves alterations of basic loss functions. For instance, the complex wavelet loss function in the first row of Table A1 is a modified Charbonnier loss function. Importantly, the rest of the loss functions make use of the $\ell_{1}$, $\ell_{2}$ and Frobenius norms, respectively.

**Table A1:** Listing of loss functions used by desnowing methods.

Loss Function	Ref.	Description
$\sum_{i}^{}\sqrt{\left|\right|\left({\tilde{\eta }}_{i}^{\pi }-\eta_{i}^{\pi }\right)+j\left({\tilde{\eta }}_{i}^{\pi }-\eta_{i}^{\pi }\right){|}^{2}+\epsilon^{2}}$	Chen et al. [30]	Complex Wavelet Loss
$||CC\left(J\right)-CC\left(J_{GT}\right){\left|\right|}_{1}$	Chen et al. [30]	Contradict Channel Loss
$\frac{1}{C_{j}H_{j}W_{j}}\left|\right|\phi_{j}\left(\tilde{y}\right)-\phi_{j}\left(y\right){\left|\right|}_{2}^{2}$	Chen et al. [30]	Perceptual Loss
$\sum_{i=0}^{\tau }\left|\right|P_{2^{i}}\left(m\right)-P_{2^{i}}\left(\tilde{m}\right){\left|\right|}_{2}^{2}$	Liu et al. [29]	Lightweight Pyramid Loss
$\frac{1}{N}\sum_{i=1}^{N}\left|\right|x_{i}-f\left(x_{i}\right){\left|\right|}_{2}$	Liu et al. [29]	Total Variation Loss
$\left|\right|G_{j}^{\phi }\left(\tilde{y}\right)-G_{j}^{\phi }\left(y\right){\left|\right|}_{F}^{2}$	Liu et al. [29]	Style Loss
